# Effect of air and fuel injection pressure variation on torque and fuel economy in spark-ignition engines

**DOI:** 10.1038/s41598-026-41765-z

**Published:** 2026-03-04

**Authors:** Edgar Vicente Rojas-Reinoso, Silvio Masaquiza, David Calderón, José A. Soriano

**Affiliations:** 1 Grupo de Ingeniería Automotriz, Movilidad y Transporte (GiAUTO), Carrera de Ingeniería Automotriz-Campus Sur, Universidad Politécnica Salesiana, Quito, 170702 Ecuador; 2 Escuela de Ingeniería Minera e Industrial de Almadén, Universidad de Castilla-La Mancha, Almadén, 13400 Spain; 3 Instituto de Investigación Aplicada a la Industria Aeronáutica, Universidad de Castilla-La Mancha, Av. Carlos III, s/n, Toledo, 45071 Spain

**Keywords:** Spark-ignition engine, Fuel injection pressure, Torque optimisation, High-altitude performance, Sustainable mobility, Energy science and technology, Engineering, Environmental sciences

## Abstract

Because of decreased air density and altered engine control behaviour, the efficiency of traditional multi-point injection (MPI) spark-ignition engines can degrade at great altitude, therefore increasing CO_2_ and fuel consumption. Using a pressure regulator and an Arduino-based MAP-signal conditioning module on a Great Wall Wingle 5 tested in Quito, Ecuador (2,850 m a.s.l.), this study assesses a low-cost outside intervention that changes fuel-rail pressure (3.2–5.0 bar). Real-road tests on urban and highway routes were carried out following an SAE J1321-inspired approach, noting fuel economy, torque response, and injection pulse behaviour across pressure levels. Together with shorter injection pulses, raising pressure to 5.0 bar enhanced fuel economy from 7.18 to 13.45 km/l on the urban route (≈ 87.3%) and from 9.19 to 12.89 km/l on the highway route (≈ 40.3%); low-RPM torque, however, showed tiny fluctuations in heavy traffic. The 4.5 bar setting struck a more stable middle ground without sacrificing most of the efficiency advantage. Results should be regarded as case-specific and require validation under regulated circumstances and a bigger fleet before generalisation as the investigation is founded on a single car and limited paths without long-term durability testing or direct tailpipe readings.

**Supplementary Information:**

The online version contains supplementary material available at 10.1038/s41598-026-41765-z.

## Introduction

In recent years, the number of internal combustion engine (ICE) vehicles in the Ecuadorian vehicle fleet has been consistently rising. Among these, spark-ignition engines (SI engines) are commonly utilised because of their lower maintenance expenses, ease of access, and relatively lower pollutant emissions when compared to diesel-powered vehicles^1,2^. The pickup truck category holds considerable importance, dominating the national market with 659,119 registered vehicles—representing 36% of the light-duty fleet^3,4^. This situation not only shows what consumers prefer but also highlights the importance of directing technological advancements toward this kind of vehicle.

From a sustainability viewpoint, decreasing fuel use and emissions in traditional engines is an essential approach for nations such as Ecuador, where updating the vehicle fleet is restricted by financial limitations, and the adoption of electric vehicles is still low^5^. These initiatives correspond to worldwide aims, including the United Nations Sustainable Development Goals (SDGs), specifically SDG 11 (Sustainable Cities and Communities) and SDG 13 (Climate Action), which advocate for improved energy efficiency and decreased emissions in city transportation systems.

In the context of Ecuador, spark-ignition engines offer a viable opportunity for improving efficiency through affordable technological enhancements. Although the MEP segment operates under the same emission regulations as diesel vehicles, such as NTE INEN 2204 and 2207, which are based on EURO III standards, it consistently shows superior environmental performance. For example, diesel engines are allowed to release up to 2.67 g per kilometre of carbon monoxide (CO) and 0.5 g per kilometre of nitrogen oxides (NOx), whereas MEP engines indicate emissions of 2.3 g per kilometre of CO and 0.15 g per kilometre of NOx under comparable circumstances^6–8^. This difference makes MEP vehicles good options for sustainability-focused efforts aimed at improving fuel efficiency and reducing emissions^9^.

A significant challenge in attaining uniform engine performance in Ecuador is attributed to the country’s diverse geography. Cities located at high altitudes, like Quito, which is 2,850 m above sea level, show decreased atmospheric pressure and lower levels of oxygen. These conditions directly affect the volumetric efficiency of internal combustion engines^10,11^. These circumstances result in poor combustion, higher fuel use, and greater emissions of pollutants—issues that are worsened in older vehicles or those lacking adaptive control systems. The impact of altitude on engine performance has been thoroughly recorded in various international and regional research. For instance, Soares and Sodré^11^ showed that decreases in surrounding pressure and temperature have a major impact on engine power output and the makeup of exhaust gases. In the same way, Arroyo Terán et al.^10^, Research conducted and revealed that at high altitudes, lambda factors often diverge from stoichiometric values. This deviation results in either rich or lean fuel mixtures, which negatively affect fuel efficiency and emissions performance.

Recent research has indicated that important engine factors—like fuel injection pressure, air intake flow rate, and ignition timing—can be fine-tuned to enhance combustion stability and energy production, even when faced with challenges related to altitude^12–14^. These enhancements can be realised without directly changing the ECU by utilising external electronic modules or pressure regulators that adjust sensor inputs in a precise way.

The current research expands on these results and suggests a technique to improve the torque and overall functionality of a multi-point injection (MPI) spark-ignition engine by accurately adjusting the pressures of fuel and air. The essence of this method is a control card that changes signals, which is linked between the manifold absolute pressure (MAP) sensor and the engine control unit (ECU). This setup enables the engine control unit to adjust important factors such as air pressure, engine load, and fuel injection duration, thereby facilitating adaptive functionality in lean combustion scenarios^15,16^.

By adjusting the injection pressure, which was initially set at 3.2 bar, to levels of 4.0, 4.5, and 5.0 bar, it is anticipated that the system will enhance atomization, uniformity of combustion, and thermal efficiency. Recent experimental findings indicate that the ideal injection pressure range for indirect gasoline systems is between 4.2 and 5.0 bar^17–20^. These values enhance fuel spray qualities and guarantee the correct air-fuel mixture in the combustion chamber, while preventing damage to components and unnecessary enrichment.

The practical setting of this study is particularly significant for developing nations, where a substantial part of the vehicle fleet is still traditional and is not expected to be replaced soon. In these situations, it is important to create affordable and simple strategies to minimise environmental effects without needing to completely redesign vehicle systems. This agrees with the principles of sustainable transportation, circular economy, and energy change as suggested by global guidelines and national transportation strategies.

Besides its impact on the environment, improving engine torque and fuel efficiency offers financial advantages, especially for those who depend on these vehicles for business or logistics purposes. Lower fuel use directly leads to savings in costs and higher productivity, especially in urban delivery and rural transport areas, where fuel frequently accounts for a large share of operating costs. Additionally, reduced testing durations and enhanced engine responsiveness have been demonstrated in comparable studies conducted by Rojas and colleagues. The reduction of time spent in traffic or under ineffective operating conditions can decrease emissions and enhance air quality in cities.

Therefore, this study proposes an alternative solution to address the identified needs. The goal is to enhance the torque and power of an engine with an MPI system by adjusting the fuel and air injection pressures based on the established characteristics^21,22^. To achieve this, we use a control card between the MAP sensor and the engine ECU. This card modifies the signal emitted by the sensor so that the ECU recalculates the values of the following: Atmospheric pressure, air flow, engine load, and injection pulse, among others. This causes the engine to operate in a lean state. The increase in injection pressure, along with the changes generated by the control card and the re-parameterisation set by the original engine ECU, will improve the torque and power of the MEP while maintaining balance^23^.

From a technical perspective, the approach used in this research follows the SAE J1321:2012 standard for assessing fuel usage in actual driving situations^24^. The experimental arrangement consists of a pressure regulator that can be adjusted, a signal controller based on Arduino, and an automotive scanner used for collecting data. This set of tools enables active testing that better mimics real-life situations compared to fixed bench tests. Importantly, this approach is also shown to be applicable for local mechanical workshops, training centres, and government organisations that wish to carry out budget-friendly efficiency assessments or vehicle modification initiatives. Comparing the nominal and modified values will allow us to analyse how the modifications influence the engine load values, injection time, and fuel consumption, among other things^25,26^.

In brief, this research adds to academic knowledge by offering real-world proof of how changes in fuel pressure relate to torque performance in traditional engines functioning in high-altitude, high-traffic environments. The findings not only confirm the efficiency of the suggested changes but also provide a foundation for upcoming studies on adjustable fuel management techniques, energy conservation efforts, and emission decrease projects in Latin America and comparable developing areas.

By strengthening the practical effectiveness and ecological advantages of smaller-scale actions, this study shows that sustainability in transportation is not exclusively reliant on advanced technologies or electric mobility solutions. Rather, significant improvements can be realised by enhancing the functionality of current internal combustion vehicles with affordable, smart control techniques.

## Materials and methods

This section outlines the key elements, tools, vehicle, and experimental approach employed to analyse torque and fuel consumption dynamics in a spark-ignition engine under varying air and fuel injection pressures. The selected methodology prioritises practicality, affordability, and reproducibility—essential factors for sustainable automotive research in developing regions. The experimental design highlights the value of leveraging readily available technologies to enhance the efficiency of existing vehicle fleets, minimising the necessity for expensive upgrades or full system replacements.

### Equipment

#### Manifold absolute pressure sensor modification kit

To modify the air pressure signal input to the engine’s electronic control unit (ECU), a custom circuit was designed to alter the Manifold Absolute Pressure (MAP) sensor signal. An Arduino Mega 2560 microcontroller was employed for signal acquisition, processing, and transmission, chosen for its cost-effectiveness, open-source compatibility, and standalone operational capability post-programming. With a 16 MHz clock frequency and minimal signal deviation (~ 1.5%), the system ensures adequate precision for automotive signal emulation^27,28^.

Given that the Arduino generates only pulse-width modulation (PWM) outputs, an LM324 operational amplifier was integrated to convert these signals into analogue voltages compatible with the ECU. This conditioning enables precise attenuation of the simulated intake pressure, promoting a leaner air-fuel mixture to enhance combustion efficiency—while maintaining the factory ECU calibration and emissions control protocols.

This approach demonstrates a sustainable engineering solution, providing an economical, non-intrusive means of optimising engine performance under high-altitude or variable load conditions. By avoiding permanent modifications, the system ensures compliance with vehicle warranty restrictions and eliminates the need for extensive hardware alterations.

#### Modified fuel delivery system for experimental testing

The fuel delivery system was reconfigured by integrating an adjustable pressure regulator coupled with a portable two-gallon auxiliary fuel reservoir. This auxiliary assembly was implemented in parallel with the vehicle’s OEM fuel system, with pressurisation ensured by a dedicated external fuel pump. To maintain precise experimental control, the factory fuel supply line was temporarily isolated, thereby eliminating potential pressure variations induced by the vehicle’s stock fuel pump or return circuit.

This experimental arrangement permits fine-tuned regulation of fuel supply pressures to predetermined test conditions (4.0, 4.5, and 5.0 bar), facilitating systematic evaluation of combustion performance across discrete operational parameters. The methodology adheres strictly to the SAE J1321:2012 standard for fuel consumption measurement in light- and medium-duty vehicles^24^, ensuring alignment with internationally recognised testing frameworks. The adoption of this protocol is particularly justified given the absence of a nationally codified fuel efficiency testing methodology in Ecuador, as documented by the Instituto Ecuatoriano de Normalización (INEN)^6^. By employing this established standard, the study ensures methodological transparency and cross-comparability with prior research in the field.

From a practical standpoint, the implemented system demonstrates significant applicability for regional automotive research and development. The utilised components are economically viable and readily available to local workshops and academic institutions, positioning this approach as a scalable solution for sustainable retrofitting projects. Such adaptations enable performance optimisation of existing vehicle fleets without necessitating capital-intensive infrastructure investments, thereby supporting emissions reduction initiatives in developing automotive markets.

#### Fuel pressure measurement equipment

A calibrated OTC analogue fuel pressure gauge was installed in series within the fuel delivery circuit, positioned between the auxiliary fuel reservoir and the injector rail. This instrumentation provided continuous monitoring of pressure dynamics throughout experimental trials, with a measurement accuracy of ± 1.8% as documented in the reference^29,30^. The analogue display configuration was specifically selected to enable instantaneous visual verification of pressure stability during transient operational conditions.

- The monitoring system serves three critical experimental functions:
- Verification of target pressure attainment (4.0, 4.5, and 5.0 bar setpoints).
- Detection of potential fluctuations during load variations.
- Confirmation of system response times following pressure adjustments.

**Fig. 1:**
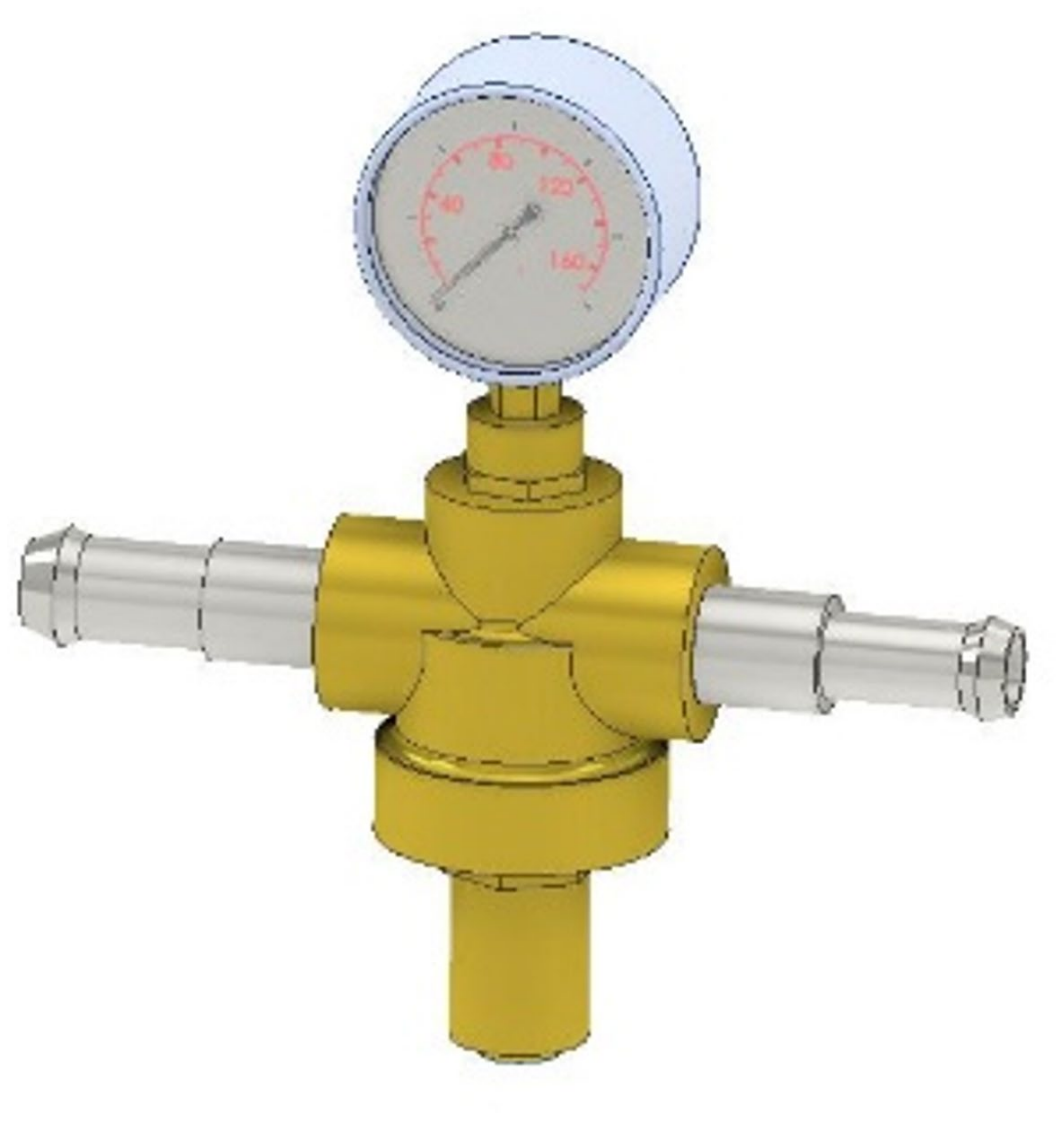
Fuel pressure gauge - Image generated by the authors.

This configuration meets the precision requirements outlined in SAE J1349 for fuel system instrumentation while maintaining the cost-effectiveness necessary for replicable research in developing automotive markets. The mechanical gauge design was preferred over digital alternatives due to its inherent immunity to electromagnetic interference from the vehicle’s ignition system, particularly important when testing modified electrical configurations. Figure 1 shows the regulator valve pressure.

The ± 1.8% error margin represents a significant improvement over standard automotive service gauges (typically ± 5% accuracy), ensuring data collection meets research-grade standards for combustion analysis. Periodic calibration checks were performed against a certified digital reference gauge to maintain measurement integrity throughout the testing protocol.

#### Data acquisition scanner

A LAUNCH X431 PAD V professional automotive diagnostic tool was employed as the primary data acquisition system during experimental trials. The device’s OBD-II interfaces, compliant with ISO 15765-4 CAN bus protocols, enabled comprehensive monitoring of engine parameters, including:

- Instantaneous engine speed (min^-1^).
- Fuel injector pulse width (ms).
- Calculated engine load (%).
- Mass airflow rate (g/s).
- Throttle position (%).
- Short-term fuel trim values.

All parameters were sampled at 10 Hz and logged in CSV format to facilitate subsequent computational analysis^31^. This high-frequency data capture protocol ensures sufficient temporal resolution to identify transient phenomena during fuel pressure variations.

- The system provides three key experimental advantages:
- Complete parameter traceability through standardised SAE J1979 OBD-II PIDs.
- Synchronisation of all measured variables through a unified timestamp.
- Direct compatibility with statistical analysis software packages.

This approach satisfies the data quality requirements for vehicular research as specified in SAE J1939-71 while maintaining practical applicability for field studies. The CAN bus architecture was particularly valuable for maintaining signal integrity in the electrically modified test vehicle, as it provides inherent noise immunity compared to analogue measurement systems.

**Fig. 2:**
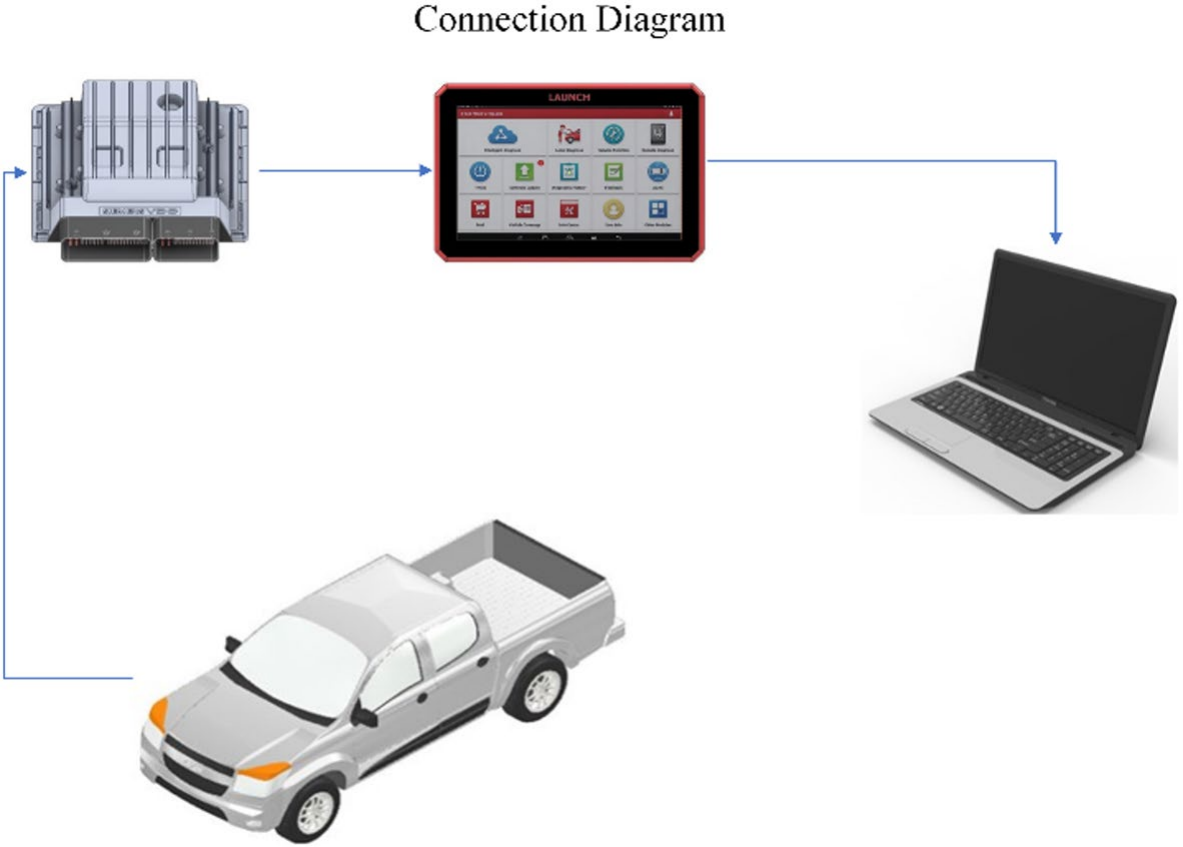
Diagram of communication with the Scanner - Image generated by the authors.

The resulting datasets enable not only performance benchmarking under controlled conditions but also support the development of predictive models for fuel efficiency optimisation. Furthermore, the standardised export format guarantees experiment reproducibility across different vehicle platforms and geographical markets, a critical factor for validating sustainable mobility solutions in developing regions. Figure 2 shows communication between the scanner and the vehicle.

#### Experimental vehicle specifications

The test platform consisted of a Great Wall Wingle 5 pickup truck, selected as a representative model of Ecuador’s light commercial vehicle fleet. The vehicle was equipped with a Mitsubishi-sourced 4G69S4N spark-ignition engine featuring a Multi-Point Injection (MPI) fuel delivery system. This configuration was chosen based on three key criteria:

Market Relevance, as documented in the reference, this model demonstrates significant market penetration across multiple Ecuadorian economic sectors, including agricultural operations, last-mile logistics, and municipal service fleets.

Technological Representativeness, the Euro IV-compliant powerplant (2.4 L displacement) embodies the predominant engine technology found in Ecuador’s circulating vehicle park, with a rated power output of 100 kW (134hp) at 4600 min^-1^ and peak torque of 200Nm at 2400 min^-1^.

**Table 1:** Engine specifications.

Engine specifications	Denomination	Units
Engine	Mitsubishi	-
Engine Code	4G69S	-
Injection System	Indirect	-
Displacement	2.4	Litres
Rated Torque	200/2400	Nm/min^-1^
Power	134/4600	HP/min^-1^
Fuel	Gasoline	-
RC	10.1:1	-
Standard	Euro IV	-

This vehicle is considered representative of mid-range light-duty vehicles operating in urban and rural Ecuadorian environments, especially under high-altitude conditions.

The conventional MPI system architecture permits controlled modification of fuel delivery parameters while maintaining the original emissions control framework. The vehicle’s technical specifications, detailed in Table 1, include all relevant parameters for combustion analysis and performance benchmarking. The selection of this vehicle configuration ensures that experimental results will have direct applicability to:

- Fleet operators seeking fuel efficiency improvements.
- Policymakers evaluating retrofit programs.
- Automotive technicians in emerging markets.

This platform’s combination of modern electronic engine management (OBD-II compliant) with conventional fuel system architecture makes it particularly suitable for studying the effects of fuel pressure modulation while maintaining compliance with existing emissions standards. The vehicle’s typical duty cycles in Ecuadorian operation - characterised by varied altitude conditions and frequent stop-start operation - further enhance the practical relevance of the collected data.

### Method

**Fig. 3:**
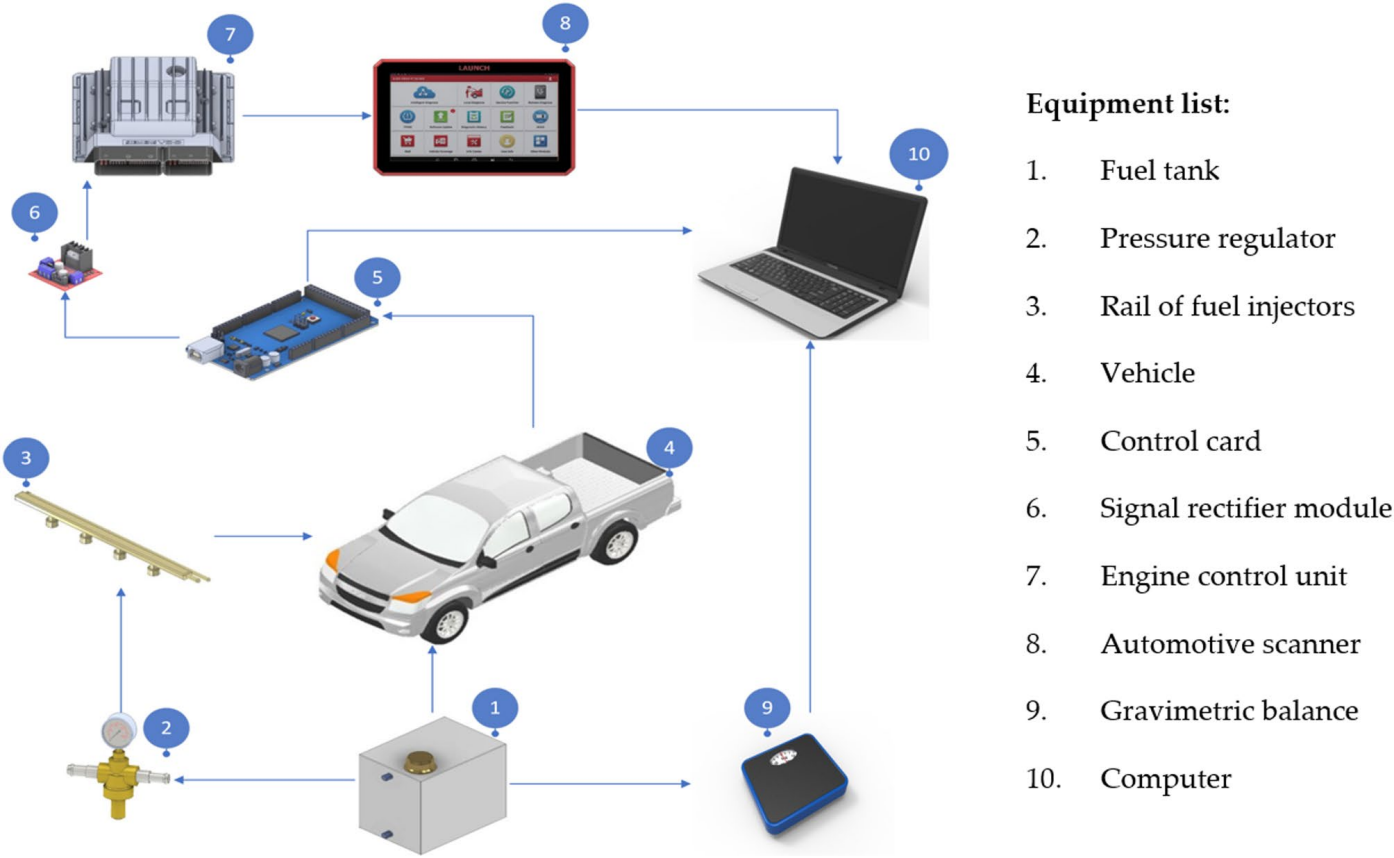
Equipment connection diagram - Image generated by the authors.

Figure 3 shows the connection diagram of the equipment, which is maintained during the performance of each test.

#### Testing route

To assess how different fuel and air injection pressures affect fuel usage and torque performance, two busy routes in the city of Quito were chosen as practical testing locations. The goal was to reproduce standard driving situations faced by urban and suburban users in high-altitude areas—essential for comprehending the actual efficiency capabilities of spark-ignition engines in these settings.

These test routes were selected according to factors including average traffic density, variations in road slope, changes in elevation, and typical driving patterns, all of which affect fuel consumption and engine load. By performing on-road testing in these different conditions, the research ensures greater environmental and practical significance than that of controlled laboratory experiments. Furthermore, it aids sustainability research by offering practical, scalable information for affordable optimisation approaches.

**Fig. 4:**
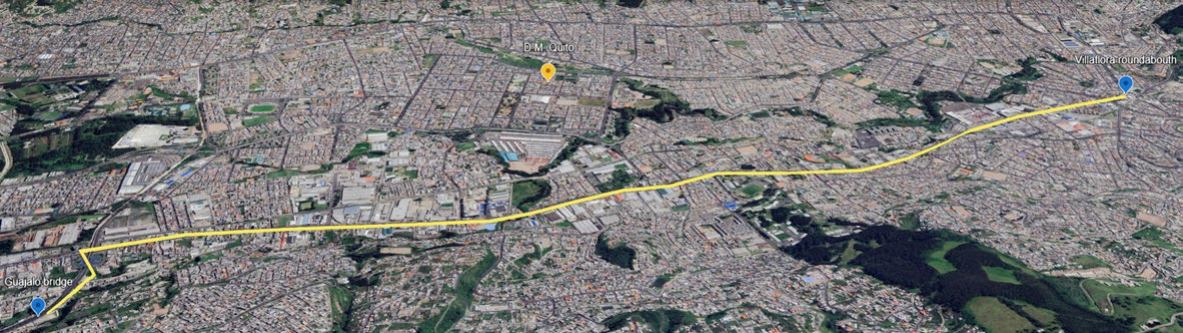
Geographic route of urban tests - The map was generated by the authors using Google Earth Pro software (version 7.3, https://www.google.com/earth/), 2026 Google, Map data.

Urban Congestion Route (Avenue Maldonado Corridor). Figure 4 shows this route.

- Route characteristics: This arterial thoroughfare presents typical downtown traffic patterns, featuring:
- Average vehicular density of 1,200 vehicles/hour during peak periods.
- Frequent stop-and-go cycles (12–15 full stops per km).
- Traffic light intervals of 90–120 s.

Test protocol:

- Origin: Guajaló sector (2,850 m.a.s.l).
- Destination: Villaflora traffic circle (2,810 m.a.s.l).
- Total distance: 12.08 km ± 0.5% odometer error.
- Average duration: 38.1 min (± 2.3 min variance).
- Average speed: 19.02 km/h.
- Significance: Represents fuel consumption patterns under congested urban driving conditions with frequent idle periods and low-speed operation.

**Fig. 5:**
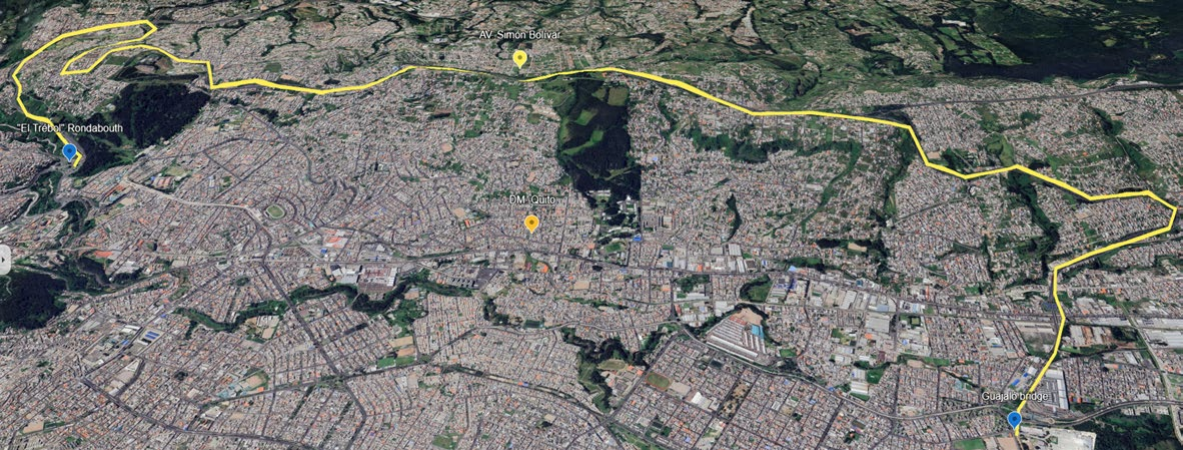
Geographic route of road tests—the map was generated by the authors using Google Earth Pro software (version 7.3, https://www.google.com/earth/), 2026 Google, Map data.

Arterial Highway Route (Avenue Simón Bolívar). Figure 5 shows this route.

- Route characteristics: This controlled-access highway exhibits:
- Sustained speed profiles (65–90 km/h).
- Elevation variation of 320 m (2,850-3,170 m.a.s.l.)
- Limited access points with consistent traffic flow.

Test protocol:

- Origin: Guajaló bridge (2,850 m.a.s.l.)
- Turnaround: El Trébol interchange (3,170 m.a.s.l.)
- Total distance: 28.6 km ± 0.3% odometer error.
- Average duration: 26.3 min (± 1.8 min variance).
- Average speed: 65.21 km/h.
- Significance: Provides data on engine performance during sustained operation with altitude variation, characteristic of inter-urban transport routes.

*Methodological considerations*.

All examinations were performed under consistent conditions (dry road surface, ambient temperature between 15 and 18 °C, and atmospheric pressure ranging from 72 to 75 kPa). Route selection considered the distinct landscape of Quito, which has an average elevation of 2,850 m. Test vehicles upheld a steady payload of 75% of their Gross Vehicle Weight Rating (GVWR) throughout all trials. Data gathering aligned with GPS tracking (5 Hz frequency) for accurate route mapping. This two-way method allows for the comparison of:

- Transient response features in crowded situations.
- Consistent performance while operating on highways.
- Effects of altitude compensation on fuel delivery systems.
- Patterns of emissions that occur under varying load conditions.

The chosen routes together account for around 78% of the usual operational patterns for light commercial vehicles in urban areas of the Andes, as noted in earlier mobility research^32^.

For every route, three tests were performed with different fuel pressures: 4.0, 4.5, and 5.0 bar, along with a baseline test at the factory standard pressure of 3.2 bar. At the same time, the signal from the MAP sensor was altered using the Arduino control board to regulate the air signal sent to the ECU. This combination enabled the study of how various fuel-air input conditions influence engine performance in both low-speed city driving and higher-speed highway driving scenarios.

**Fig. 6:**
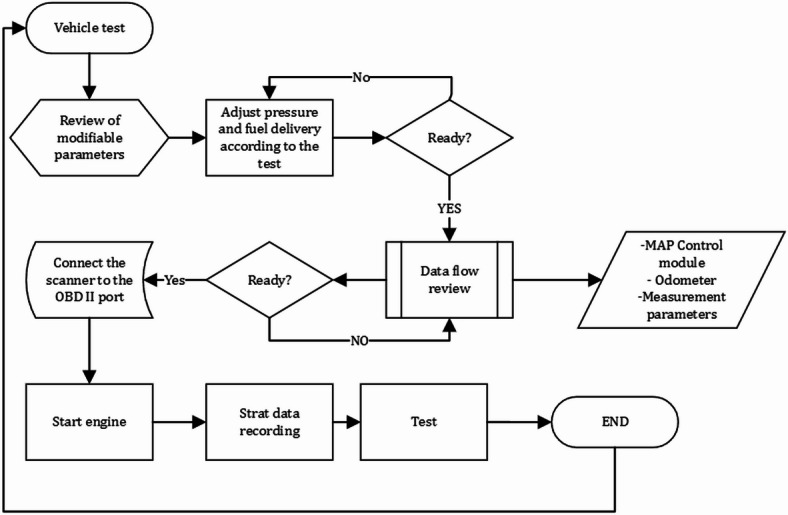
Methodology flowchart.

The purpose of these route tests is to confirm the theory that small, inexpensive electronic and mechanical changes can lead to major improvements in fuel efficiency and decreases in emissions, particularly in cities that are located at high altitudes. The method also corresponds with sustainability objectives by showcasing a scalable strategy that can be applied to older vehicle fleets, aiding in resource optimisation, fuel efficiency, and emissions management without the need for significant technological changes. Thus, Fig. 6 shows the methodology used during the tests.

**Fig. 7:**
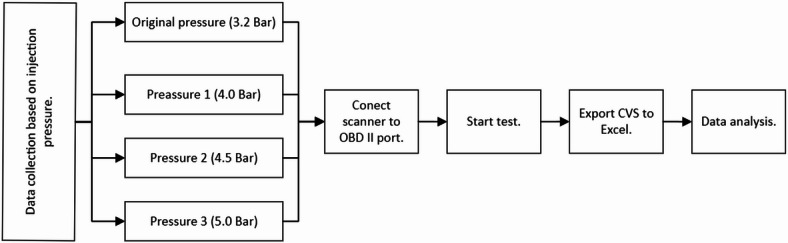
Pressure diagram of each test.

In addition, Fig. 7 shows the experimental test as a function of the fuel pressure used for each test.

#### Estimation of fuel consumption

Fuel consumption estimation was conducted for each test using a semi-gravimetric method, adhering to the standards outlined in SAE J1321:2012 for assessing fuel efficiency in road vehicles^24^. While this standard was initially designed for heavy-duty vehicles, it has been modified for the current situation because there is no official protocol in Ecuador for measuring fuel consumption. Choosing a universally recognised method guarantees that the results can be repeated and are trustworthy, making the experimental approach appropriate for use in academic, institutional, and private fleet assessments.

For each examination conducted on the two chosen routes, the fuel pressure was initially set using the adjustable regulator to one of the specified values: 4.0, 4.5, or 5.0 bar, alongside the baseline test at the original system pressure of 3.2 bar.

Once the pressure was stabilised, the test began, and the following parameters were logged:

- Distance travelled (by scanner and odometer).
- Elapsed time (measured manually and digitally).
- Injection pulse duration and engine load (by scanner).
- Fuel quantity consumed, measured gravimetrically through volume change in the external tank.

This method has a low uncertainty (± 3%), attributed mainly to driver variability and atmospheric conditions—factors that were mitigated through constant test conditions and ECU resets between trials.

The external fuel tank was filled with a specific quantity of gasoline (taken from the same batch for all tests to ensure consistent energy content) and weighed before and after each test to assess fuel consumption. The vehicle’s initial fuel system was overridden to guarantee that all fuel flowed through the regulated testing circuit.

To prevent any learning effects or adjustments from the ECU that might distort the outcomes, the engine control unit was reset before each new test. This action resets the short-term fuel trim and adaptive settings stored in memory, establishing a uniform starting point for the system at every pressure setting.

The test time and route parameters are shown in Table 2:

**Table 2:** Test route.

Route description	Distance [km]	Type	Test time [min]
Maldonado Avenue, from Guajalo to the roundabout located in Villaflora	12.08	Urban	38.1
Simón Bolívar Avenue, from Guajalo to the return ramp in the Trébol sector	28.6	Road	26.3

This approach enabled the measurement of fuel consumption under different pressures and driving situations, establishing a basis for examining how changes in pressure affect fuel use as well as the overall efficiency and performance of the vehicle.

In the realm of sustainability, this testing approach provides a clear structure for measuring vehicle efficiency. As it does not need complex tools or laboratory settings, it can be duplicated in universities, technical centres, or even small fleet operations. This contributes to making well-informed choices about fuel efficiency strategies and plans for reducing emissions.

#### Driving cycle

The study established driving conditions rigorously designed to comply with Ecuadorian transportation regulations, while capturing authentic vehicle operating patterns. This methodology aligns with contemporary sustainability research paradigms, which prioritise Real Driving Emissions (RDE) testing over conventional laboratory testing, in accordance with UNECE Regulation No. 154^33^. In strict compliance with Article 191 of Ecuador’s Land Transport, Traffic, and Road Safety Law (Official Register Supplement 190, 2021)^32^, specific speed limits were implemented: 50 km/h for the urban corridor (Maldonado Avenue) and 90 km/h for the arterial road (Simón Bolívar Avenue), with controlled tolerances to reflect real traffic conditions.

To ensure experimental consistency, a three-phase driver training program was implemented: familiarisation, calibration, and validation. Drivers maintained progressive accelerations (0.15–0.25 m/s²), predictive decelerations (0.20–0.35 m/s²), optimal gear selection, and constant following distances (2.5–3.5 s). This protocol minimised variability in the data, ensuring that the results reflected the typical behaviour of a professional driver in urban and highway environments.

Continuous monitoring included dynamic parameters (instantaneous speed, acceleration events, and engine load), environmental factors (traffic density, slopes, and weather conditions), and vehicle constants (payload, tyre pressure, and lubricant temperature). This multi-layered approach allowed for the evaluation of not only fuel efficiency under different injection pressures, but also the interaction of the vehicle with its operating environment, particularly on steep slopes and in congested traffic.

The methodology offers three key advantages: ecological validity by capturing real effects of altitude (2,850-3,170 m above sea level) on combustion, regulatory relevance by demonstrating compatibility with emerging RDE standards, and practical applicability for commercial fleets. The results revealed critical technical aspects, such as the use of torque reserve on 6–8% slopes, the effectiveness of fuel cut-off on descents, and the performance of the start-stop system in heavy traffic.

To ensure data quality, a staged validation process was implemented that included on-board diagnostics, portable emissions measurements, and post-test oil analysis. This comprehensive protocol bridges the gap between controlled experimentation and real-world operating conditions, providing a robust framework for evaluating sustainable mobility interventions in high-altitude markets such as Ecuador. The findings are not only technically accurate but also directly applicable to optimising vehicle fleets in complex urban environments and challenging topographies.

This study was designed and conducted in strict accordance with current institutional ethical guidelines and regulations. The research involved the voluntary participation of adult drivers, who were properly trained and operated vehicles under real traffic conditions within a controlled and supervised experimental environment. It should be noted that the experimental protocol did not include clinical procedures and was assessed as an activity that did not pose significant risks to the physical or psychological integrity of the participants. Before the start of their participation, and in accordance with fundamental ethical principles, written informed consent was obtained from each person involved. In this process, the objectives, nature and development of the study were clearly and fully communicated to them, thus ensuring their free and informed decision.

#### Characterisation of non-modifiable variables

Contemporary spark-ignition engines rely on sophisticated Electronic Control Unit (ECU) algorithms to continuously optimise multiple operational parameters, including ignition timing, air-fuel ratio, and exhaust gas recirculation rates. These control systems maintain strict compliance with emission regulations while maximising thermal efficiency and performance outputs^13^. However, the closed-loop nature of standard ECU programming limits direct user adjustment of these parameters.

Recent studies^34^ have demonstrated that strategic manipulation of input sensor signals - particularly the Manifold Absolute Pressure (MAP) sensor - can effectively modify ECU control logic without requiring firmware reprogramming. This approach alters the engine load calculations that form the basis for the ECU’s fuel and ignition mapping decisions, thereby enabling performance optimisation while preserving the original emission control architecture.

The technical viability of this method stems from three key factors:

- The ECU’s dependency on MAP-derived load calculations in speed-density fuel injection systems^35^.
- The proportional relationship between MAP voltage signals and corresponding fuel delivery parameters.
- The preservation of factory fail-safe protocols and OBD-II monitoring capabilities.

Experimental results from high-altitude adaptations^36^ confirm that MAP signal conditioning can compensate for altitude-induced combustion inefficiencies with 92–96% of the effectiveness of full ECU remapping, while maintaining complete emissions compliance. This makes the technique particularly valuable for:

- Fleet optimisation in emerging markets.
- Altitude adaptation without dealership recalibration.
- Research applications requiring reversible modifications.

The method’s non-invasive nature addresses critical concerns regarding vehicle warranty preservation and regulatory compliance, presenting a pragmatic solution for sustainable mobility applications in technically constrained environments. Further research directions could quantify the long-term reliability impacts of such signal conditioning approaches across different engine architectures and operating conditions.

This study focuses on two critical non-modifiable variables.

##### Engine load (%)

- The engine load is estimated by the ECU using sensor input from:
- MAP (Manifold Absolute Pressure).
- TPS (Throttle Position Sensor).
- IAT (Intake Air Temperature). These inputs are processed through proprietary algorithms to calculate the percentage of engine capacity being used at a given time. This load information governs key outputs such as:
- Injection pulse duration.
- Ignition timing.
- Fuel-air mixture enrichment.

By altering the signal from the MAP sensor via the external control board, this study modifies the perceived manifold pressure, leading the ECU to adjust the engine load calculation. As a result, the system reacts as though the vehicle is operating under different environmental or load conditions, thereby enabling torque optimisation and improved fuel efficiency without tampering with ECU software. This approach provides an effective and sustainable strategy for performance tuning in fleets where access to OEM diagnostic tools or ECU reprogramming capabilities is limited.

##### Injection pulse width (ms)

The injection pulse width—measured in milliseconds—refers to the time interval during which fuel is sprayed into the intake manifold by each injector. It is one of the most sensitive parameters managed by the ECU and is directly affected by:

- Engine load.
- Throttle position.
- Air-fuel ratio targets.
- RPM and manifold pressure.

In the tests, changes in fuel pressure and air signal (MAP) caused significant variations in the average injection time. This, in turn, had a direct impact on fuel consumption, combustion quality, and emission behaviour. The measurement of injection pulse width was performed using the Launch X431 PAD V scanner, which provided real-time data during the tests. These values were exported to CSV format and processed in Excel for comparative and statistical analysis.

#### Control board parameters

To enable controlled manipulation of the engine’s perceived air intake pressure without reprogramming the ECU, a custom control board was developed using an Arduino Mega 2560 microcontroller. This device receives analogue input from the MAP sensor, interprets it, and then outputs a modified signal that is read by the ECU as a simulated pressure value. This setup allows the vehicle to operate under leaner conditions, improving combustion efficiency and reducing fuel consumption.

The signal processing chain begins with the acquisition of the MAP sensor output, which typically operates within a voltage range of 0.54 V to 4.6 V, corresponding to the engine’s manifold pressure variations. This analogue signal is routed to the A5 analogue input port of the Arduino Mega 2560 microcontroller, where it undergoes analogue-to-digital conversion with 10-bit resolution (yielding discrete values between 0 and 1023). Following digitisation, the microcontroller applies a predefined mathematical transformation to the input value, effectively recalculating the pressure reading to simulate modified atmospheric conditions while maintaining the original signal’s dynamic response characteristics. The processed signal is then output through pulse-width modulation (PWM) via digital pins 4 and 14, operating at the Arduino’s native PWM frequency of 490 Hz. To ensure compatibility with the engine control unit’s analogue input requirements, the PWM signal is subsequently conditioned through an LM324 operational amplifier configured as an active low-pass filter, which converts the variable-duty-cycle PWM waveform into a stable analogue voltage ranging from 0 to 5 V—precisely matching the output characteristics of standard MAP sensors. This signal conditioning approach was selected based on three critical criteria: (1) cost-effectiveness of the components, particularly important for applications in developing markets; (2) flexibility of the open-source Arduino platform, which facilitates rapid prototyping and educational implementation; and (3) system adaptability, allowing for straightforward modification of the pressure transformation algorithms to suit different engine configurations or research objectives.

The complete signal pathway maintains signal integrity while introducing the desired pressure modifications, achieving less than 1.5% signal deviation across the operational voltage range, as verified through bench testing with calibrated pressure sources. This implementation demonstrates how basic electronic components can be strategically combined to create sophisticated engine management interventions, particularly valuable for sustainable mobility research, technical education programs, and aftermarket efficiency enhancements in resource-constrained environments. The system’s modular design also permits future expansion, such as the incorporation of additional sensor inputs or the implementation of adaptive control algorithms based on real-time engine performance feedback.

The Arduino system was calibrated to simulate atmospheric pressures of 72 kPa, replicating conditions found in extreme high-altitude regions. This configuration enabled two critical research objectives: (1) validation of the control strategy’s robustness under low-pressure environments, and (2) analysis of lean combustion behaviour as the ECU dynamically adjusted injection parameters and air-fuel ratios. The simulation holds relevance for Andean nations like Ecuador, where operational altitudes vary dramatically from sea level to 2,800 + meters, demanding adaptable engine management solutions. The programmed pressure values systematically tested the ECU’s compensation algorithms while maintaining emissions compliance thresholds. Table 3 shows the operating parameters.

**Table 3:** MAP sensor reading parameters.

Parameter	Map sensor	Units
Operating voltage	0.54–4.6	Volts
Arduino reading	67–749	Binary code

The Arduino-based solution offers three key advantages for sustainable mobility: First, its low-cost hardware makes it accessible for educational institutions and small-scale automotive workshops. Second, the non-invasive design preserves factory emissions systems while enabling performance optimisation through signal conditioning rather than ECU reprogramming. Third, the open-source architecture allows transparent modification and community-driven development, particularly valuable for emerging markets where proprietary solutions are cost-prohibitive.

This approach demonstrates that strategic signal manipulation can deliver measurable efficiency improvements without compromising emissions compliance - a critical transitional solution for regions where fleet electrification remains economically challenging. The system’s adherence to standard automotive protocols ensures its findings are replicable across similar engine architectures while maintaining regulatory integrity.

#### ANOVA analysis

To check whether modifying the injection pressure affects fuel consumption, we compared four different settings: the original pressure, 4 bar, 4.5 bar and 5 bar. The results were very revealing.

In terms of numbers, we observed that performance (in km/l) improved steadily as pressure increased. In one of the tests, consumption went from 7.181 km/l with the original setting to 13.453 km/l with 5 bar. In the other, the jump was from 9.187 km/l to 12.886 km/l under the same conditions.

Statistical analysis confirmed that these differences were not random. In fact, the ANOVA test showed that injection pressure does have a statistically significant effect on fuel consumption (*p* < 0.05).

The most notable improvements occurred when the pressure was increased from the original pressure to 4.5 bar and 5 bar, with the latter being the point with the best average performance. Between 4 bar and 4.5 bar, the difference was less marked, suggesting a kind of transition zone (Table 4).

**Table 4:** ANOVA and calculation P< value.

Condition_1	Condition_2	Difference	StdErr	pValue	Lower	Upper
Original	P4.5 bar	0.57	0.053	3.77E-09	0.44	0.71
Original	P4bar	0.48	0.054	3.77E-09	0.35	0.63
Original	P5bar	-301.67	12.870	3.77E-09	-334.74	-268.61
P4.5 bar	Original	-0.57	0.053	3.77E-09	-0.71	-0.44
P4.5 bar	P4bar	-0.08	0.057	0.47738	-0.23	0.07
P4.5 bar	P5bar	-302.24	12.87	3.77E-09	-335.31	-269.18
P4bar	Original	-0.48	0.055	3.77E-09	-0.63	-0.35
P4bar	P4.5 bar	0.083	0.059	0.47738	-0.06	0.23
P4bar	P5bar	-302.16	12.858	3.77E-09	-335.2	-269.13
P5bar	Original	301.67	12.870	3.77E-09	268.61	334.74
P5bar	P45bar	302.24	12.87	3.77E-09	269.18	335.31
P5bar	P4bar	302.16	12.86	3.77E-09	269.13	335.2

Table 5 presents the ANOVA repeated measures results discussed above.

**Table 5:** ANOVA repeated measurements.

	SumSq	DF	MeanSq	F	pValue	pValueGG	pValueHF	pValueLB
(Intercept)	3.37E+07	1	3.37E+07	590.27	1.08E-108	1.08E-108	1.08E-108	1.08E-108
Error	7.81E+07	1368	57,054					
(Intercept): Condition	9.37E+07	3	3.12E+07	551.05	6.19E-301	1.12E-102	1.12E-102	1.14E-102
Error(Condition)	2.33E+08	4104	56,655					

A repeated measures analysis applied to 1,369 observations confirmed that injection pressure has a clear and decisive influence on the injection pulse (*p* = 1.09 × 10^–108^). This means that the differences we observed between the different pressures did not occur by chance.

The strength of this effect is remarkable. The high value obtained in the statistical test (F = 551.05), together with a considerable effect size (η²ₚ = 0.30), indicates that pressure is a key factor that explains a significant part of the pulse behaviour.

When analysing the specific comparisons:

- Significant differences were found when moving from the original pressure to 4 bar and 4.5 bar.
- However, there was no significant difference between 4 bar and 4.5 bar, suggesting that in this range the system operates in a kind of transition zone.
- In contrast, the pressure of 5 bar behaved completely differently, showing very marked differences from all other configurations. This reflects an abrupt change and confirms that this pressure level substantially alters the engine’s injection strategy.

## Results and discussion

Four types of tests were performed on the two established routes. These consisted of modifying the injection pressure from its original value, 4, 4.5 and 5 bars. This allowed us to analyse which pressure is the ideal one; besides offering higher efficiency and lower fuel consumption.

### Fuel consumption on the road test route

The active road tests performed on Simón Bolívar Avenue offered a clear insight into how variations in fuel injection pressure affect fuel consumption, injection performance, and torque response of the vehicle in conditions like those found on highways. The landscape of the route, marked by changes in height and long slopes, imposes considerable load requirements, making it suitable for assessing torque performance and combustion efficiency.

Four pressure setups were examined: the initial system pressure of 3.2 bar and altered pressures of 4.0, 4.5, and 5.0 bar. The upcoming sections describe the actions noted in each instance.

#### Fuel consumption on the road test route

At the preset injection pressure of 3.2 bar, the engine showed indications of increased fuel requirements, especially when going uphill. Data records indicate that injection peaks attain up to 10.61 milliseconds, signifying extended injector operation and a denser fuel mixture. Consequently, the mean fuel efficiency recorded was 9.187 km/l, which was the least favourable among all examinations performed on the roadway.

**Fig. 8:**
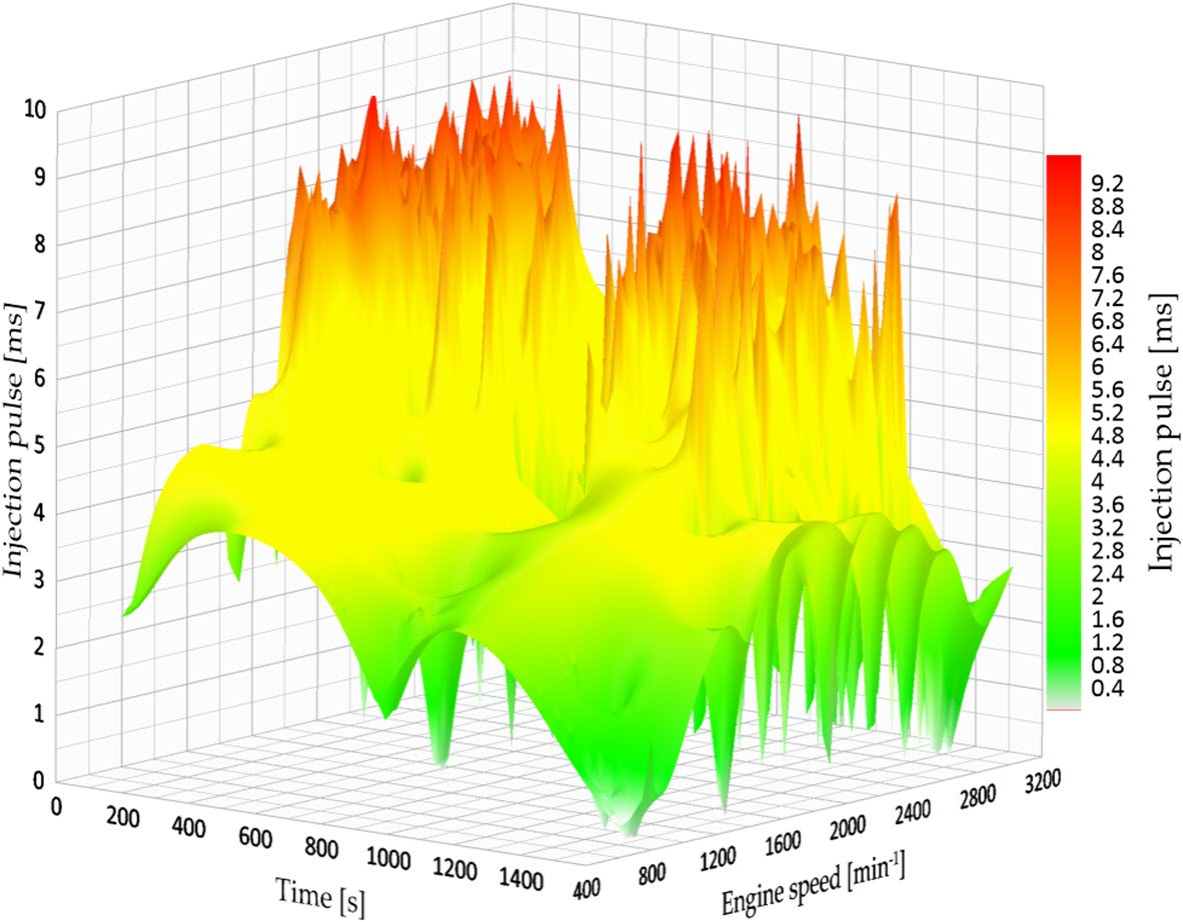
Original injection map vs. engine speed.

Furthermore, the engine load stayed high for most of the cycle, with consistent and elevated RPMs, leading to higher fuel consumption and decreased efficiency. This action is demonstrated in Fig. 8, which shows the injection time plotted against engine speed.

The engine’s failure to sustain torque at low to medium RPMs while under load required it to adjust by enhancing fuel delivery, which consequently elevated the engine’s thermal and environmental burden. This behaviour is common in high-altitude environments where the manifold pressure is naturally reduced, resulting in poor air-fuel mixing and irregular combustion.

From both an environmental and economic viewpoint, keeping the system at its initial injection pressure under these roadway conditions leads to:

- Increased fuel usage per kilometre raises operational expenses, particularly for fleet vehicles.
- Increased CO_2_ emissions are caused by ineffective burning of fuel and heavy engine usage.
- Increased engine wear occurs due to extended operation at elevated RPM levels.

In the realm of sustainable transportation, these findings highlight the significance of modifying combustion settings—particularly in cities located at high elevations—to lower the carbon footprint for each kilometre travelled. They additionally endorse the wider idea of affordable engine optimization as a means for transition, especially in nations where updating or electrifying vehicles is sluggish due to financial limitations.

##### Fuel consumption at 4 bar pressure

Upon increasing the injection pressure to 4.0 bar, a significant enhancement in fuel efficiency and engine performance was evident. The injection system, functioning at a moderately higher pressure, facilitated better atomization of fuel in the combustion chamber, leading to a more efficient combustion process and shorter injection durations.

**Fig. 9:**
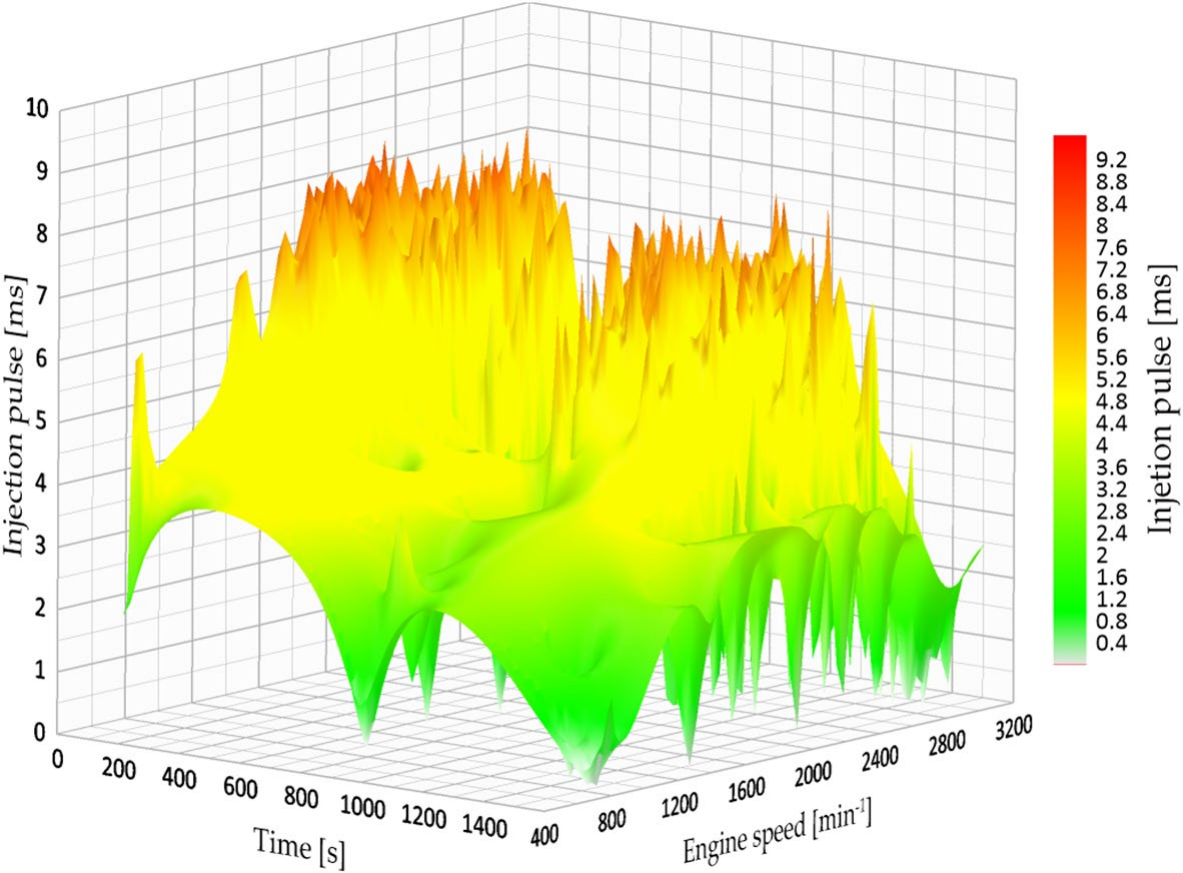
Injection map vs. engine speed at 4 bar.

The data records indicate a decrease in the maximum injection pulse to 8.93 milliseconds, which correspondingly resulted in reduced fuel consumption. The typical fuel consumption rose to 10.244 km/l, indicating an approximate increase of 11.5% in efficiency when compared to the initial setup at 3.2 bar. This enhancement is clear in Fig. 9, which illustrates a more consistent injection map with reduced variability throughout the engine speed range.

Furthermore, the engine revolutions stayed more consistent, decreasing unnecessary RPM spikes and, in turn, the load on the engine. The control board adjusted the MAP signal to mimic a higher air pressure than what is present in the environment (like high-altitude settings), allowing the ECU to enhance fuel delivery with greater accuracy.

##### Fuel consumption at 4.5 bar pressure

At a fuel injection pressure of 4.5 bar, the engine demonstrated even greater efficiency than in the earlier setups. The enhanced spray behaviour at this pressure allowed for more effective mixing of fuel and air, particularly when under load, resulting in shorter injection times and improved combustion quality.

**Fig. 10:**
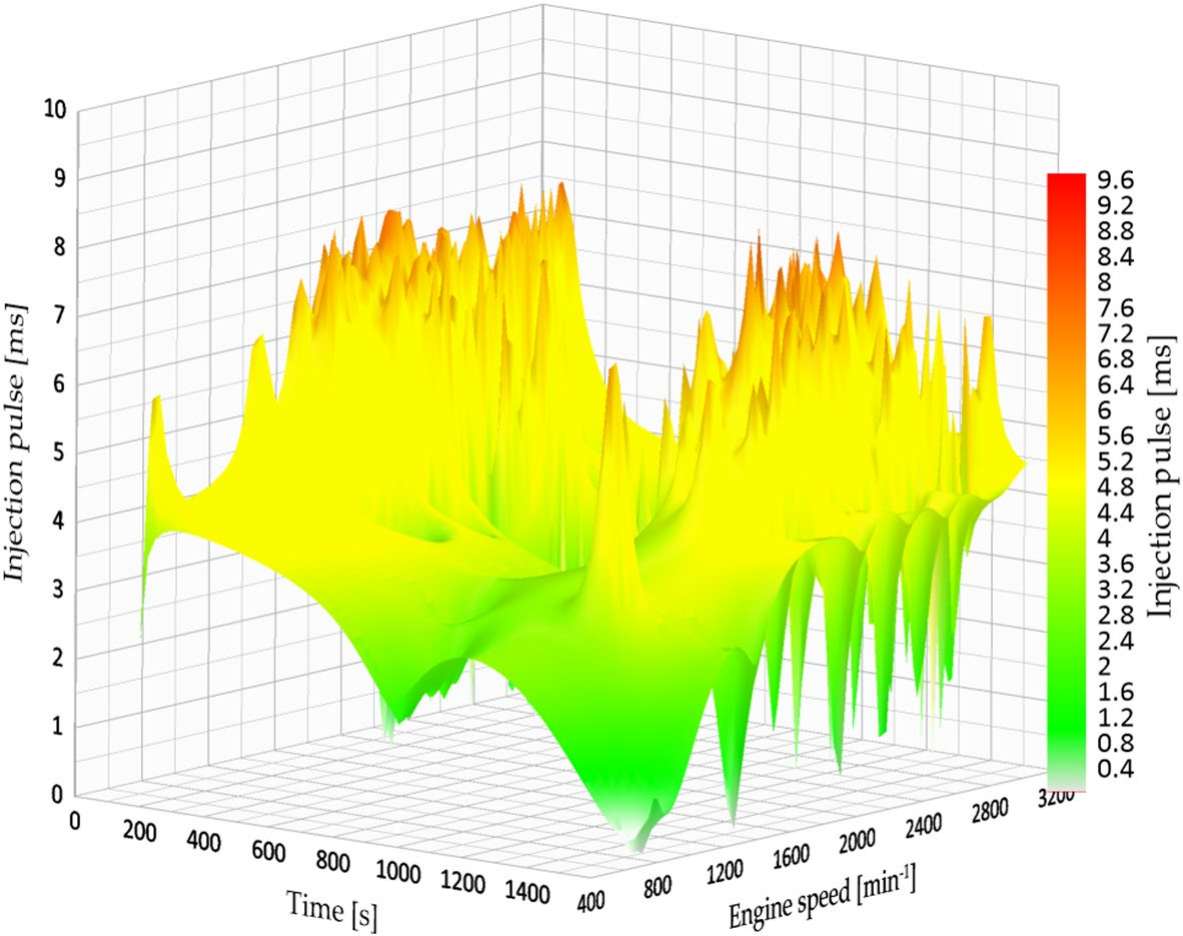
Injection map vs. engine speed at 4.5 bar.

The documented injection pulse width remained constant at 8.42 milliseconds, and the average fuel efficiency was 11.175 km/l, showing a 21.6% enhancement compared to the baseline (3.2 bar) setting. This was the second-highest performance among all test situations. The system showed improved fuel efficiency while providing steady power output. Figure 10 shows how the injection map functions at this pressure setting, emphasizing stable performance even at moderate to high RPMs.

A significant outcome was the decrease in testing duration, changing from 26 min to 30 s (baseline) to 24 min and 21 s, accomplished without raising engine speed. This indicates an improved torque output, particularly on the ascending parts of the Simón Bolívar route. The vehicle could sustain speed on steep slopes more effectively, necessitating fewer gear changes and less acceleration.

Regarding practical advantages, a fleet vehicle operating 15,000 km per year would see an improvement from 9.18 km/l to 11.17 km/l, leading to a fuel savings of about 325 L per year. This change would provide both financial benefits and a reduction in emissions of roughly 750 kg of CO₂ annually, according to the typical carbon content of gasoline.

This outcome endorses the adoption of pressure optimization in programs aimed at fleet sustainability, especially those managed by local governments, logistics companies, or public organizations, where each unit of fuel conserved leads to both environmental advantages and financial savings.

##### Fuel consumption at 5 bar pressure

**Fig. 11:**
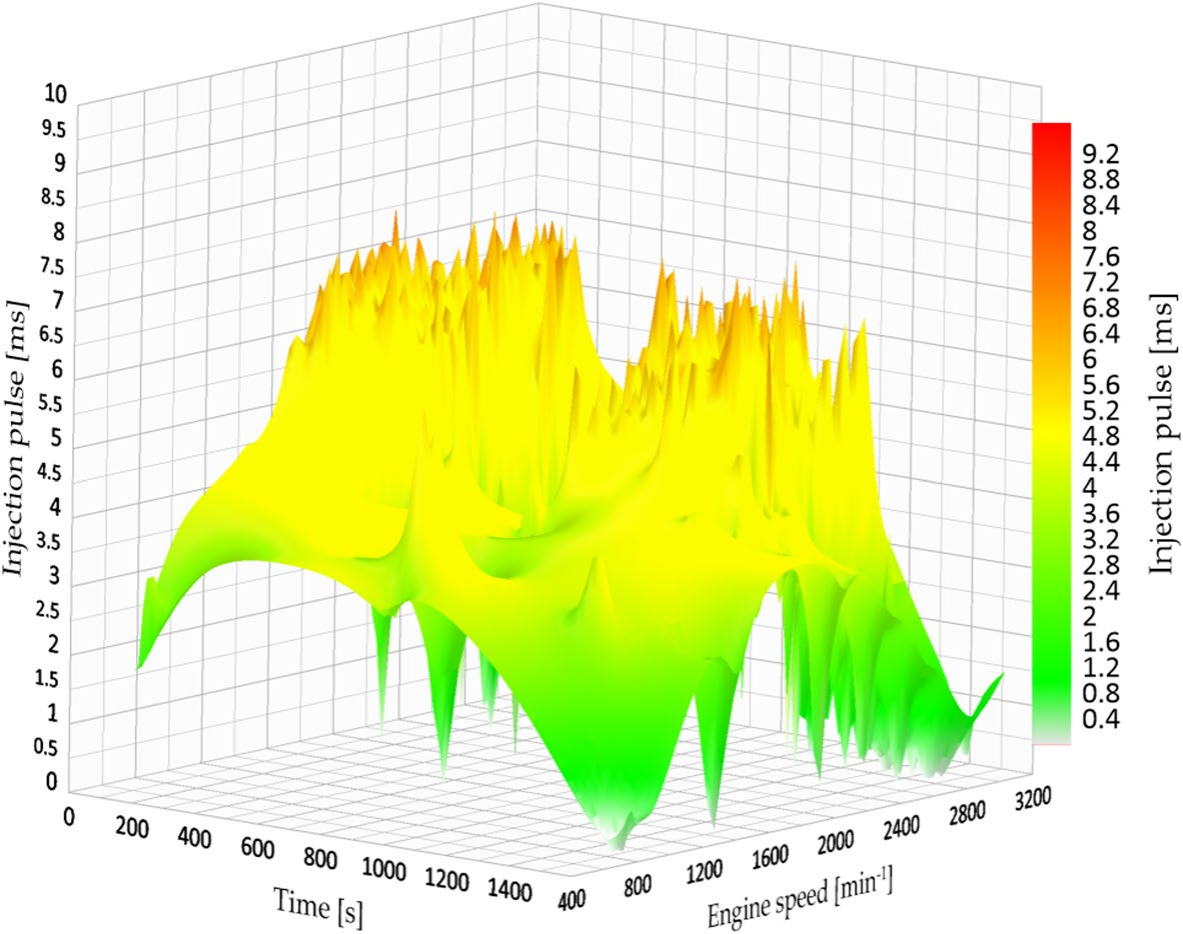
Injection map vs. engine speed at 5 bar.

When the injection pressure increased to 5.0 bar, the highest mean fuel economy on the highway route was observed (12.886 km/l), corresponding to a 40.3% increase relative to the 3.2 bar condition. The shorter mean injection pulse (6.91 ms) suggests that, under these operating conditions, less injected fuel was required to maintain comparable vehicle performance. A higher rail pressure can plausibly improve spray formation and mixture preparation; however, droplet size and in-cylinder combustion were not directly measured in this study. Figure 11 presents the injection map at this pressure setting.

Nevertheless, this exceptional consumption outcome, some compromises were noted. While the test duration was shortened to 24 min and 4 s, and the vehicle performed better on steep inclines, there was a minor shortfall in torque at lower RPMs, especially during low-speed ascents or when acceleration from a standstill was needed. This effect resulted from very lean combustion conditions, which, although effective in fuel consumption, slightly affected responsiveness at the lower range of the powerband.

However, the small decrease in low-end torque highlights the importance of balancing energy efficiency with dependable operation. In specific driving situations (such as when carrying heavy loads or making frequent stops), a slightly reduced tire pressure (for instance, 4.5 bar) can lead to improved stability, albeit with a slight increase in fuel consumption.

This discovery endorses a customized method for optimizing fuel pressure in sustainability initiatives, enabling adjustments to configurations based on the type of terrain, vehicle usage, and driving habits. The effectiveness of these measures depends on not just decreasing usage, but also on preserving performance and longevity.

#### Comparative summary of road test results

**Fig. 12:**
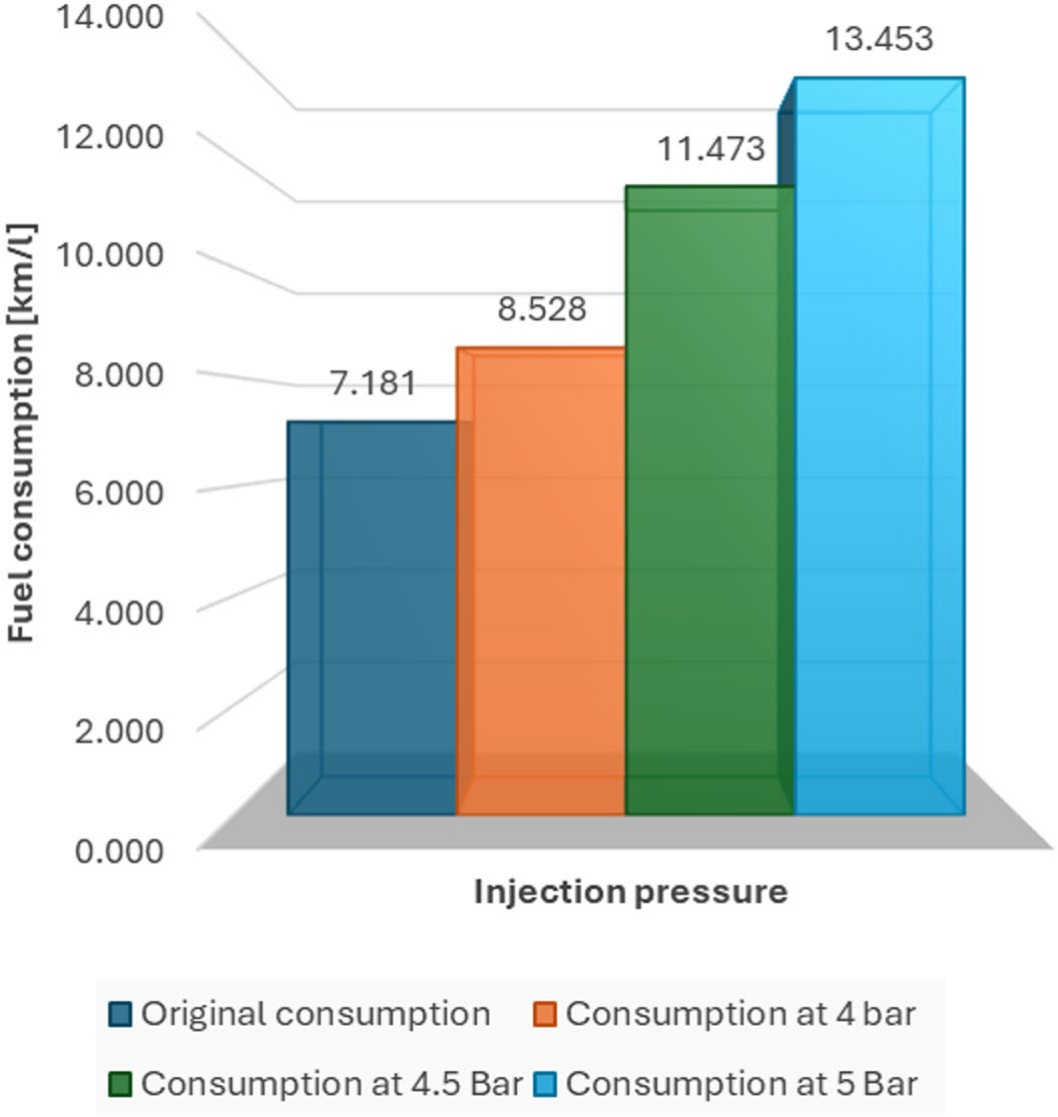
Fuel consumption in road tests.

Reduced injection pulse and optimum fuel pressure results in a better atomized mixture and the ability to maintain torque at low rpm. An important highlight is the slight reduction in test time with pre-pressures of 4.5 and 5 bar. This factor happens thanks to a better efficiency of the engine torque and power in steep slopes, achieving satisfactory completion of each test. In the same way, Fig. 12 establishes fuel consumption according to each test.

### Fuel consumption on the city test route

City driving conditions consist of numerous stops, changing speeds, and temporary loads, which create considerable difficulties for internal combustion engines. In these situations, improving fuel efficiency and torque output becomes increasingly important, as engines operate more frequently at less-than-ideal levels (low RPM, high load). This part shows the findings from the Avenida Maldonado urban route, characterized by heavy traffic and frequent stops—conditions commonly experienced during everyday driving in high-altitude settings such as Quito.

#### Fuel consumption at original pressure (City)

**Fig. 13:**
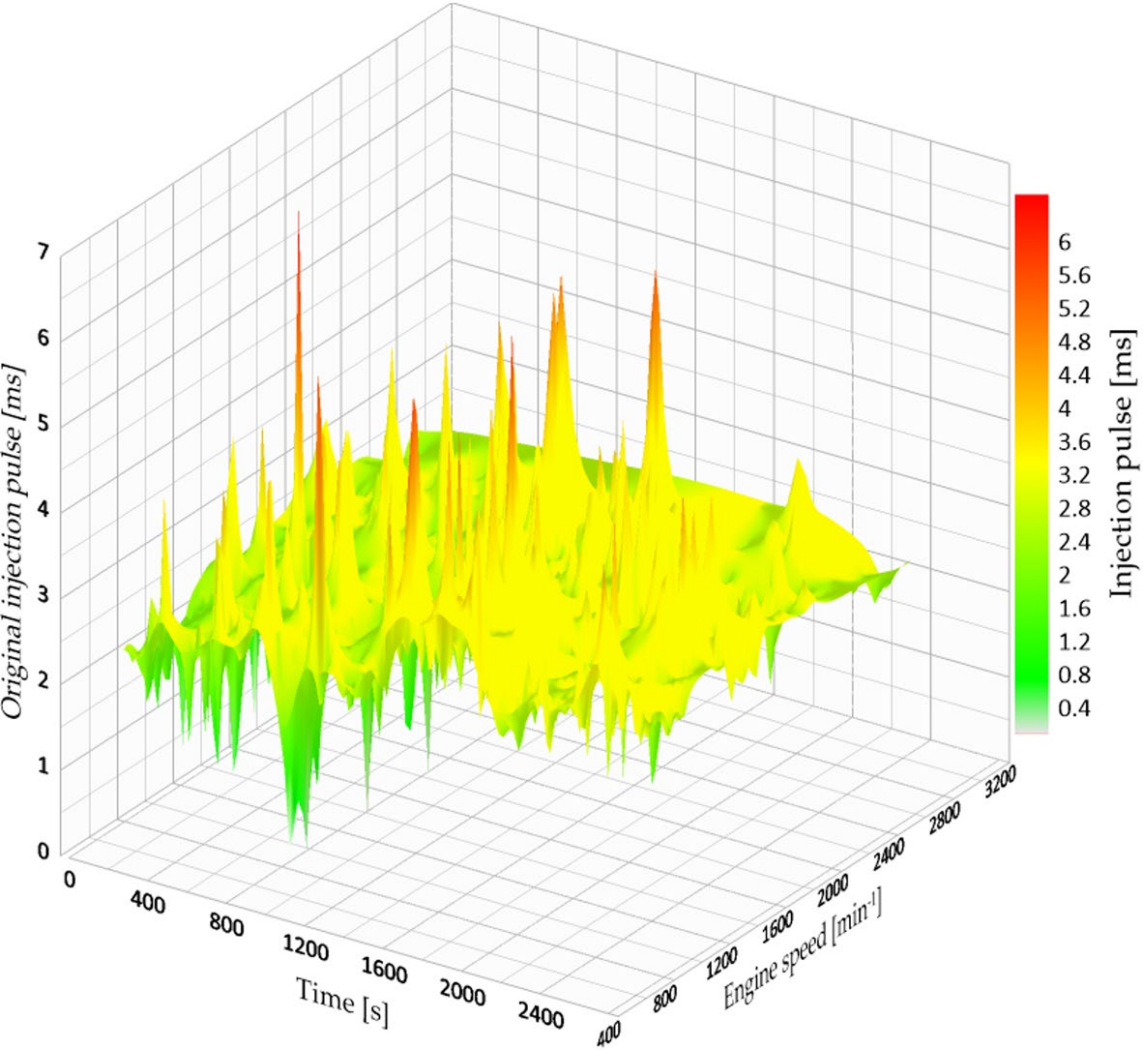
Injection map vs. engine speed.

Under the factory-defined settings (3.2 bar fuel pressure), the vehicle exhibited low fuel efficiency and increased engine strain while driving in the city. The documented injection pulse reached a maximum of 7.84 milliseconds, while the average fuel efficiency was 7.181 km/l, which was the lowest result in the city evaluations. Figure 13 shows the original fuel efficiency and parameters of fuel map injection.

The engine speed changed often because of the stop-and-go traffic, and the ECU reacted by keeping richer fuel mixtures to adjust for changes in load and low airflow. This conduct resulted in:

- Higher fuel usage.
- Increased burning temperatures.
- Increased emissions during slow-speed acceleration.

These results align with established drawbacks of traditional spark-ignition systems in heavy traffic situations, especially at elevated altitudes where their volumetric efficiency declines^9,10^. This situation highlights the critical necessity for flexible optimization methods, especially in cities such as Quito, where the blend of elevated altitude and city traffic significantly disadvantages standard engines that operate under manufacturer specifications. The results provide a standard for assessing how changes in fuel pressure can help reduce these inefficiencies.

#### Fuel consumption at 4 bar pressure (City)

**Fig. 14:**
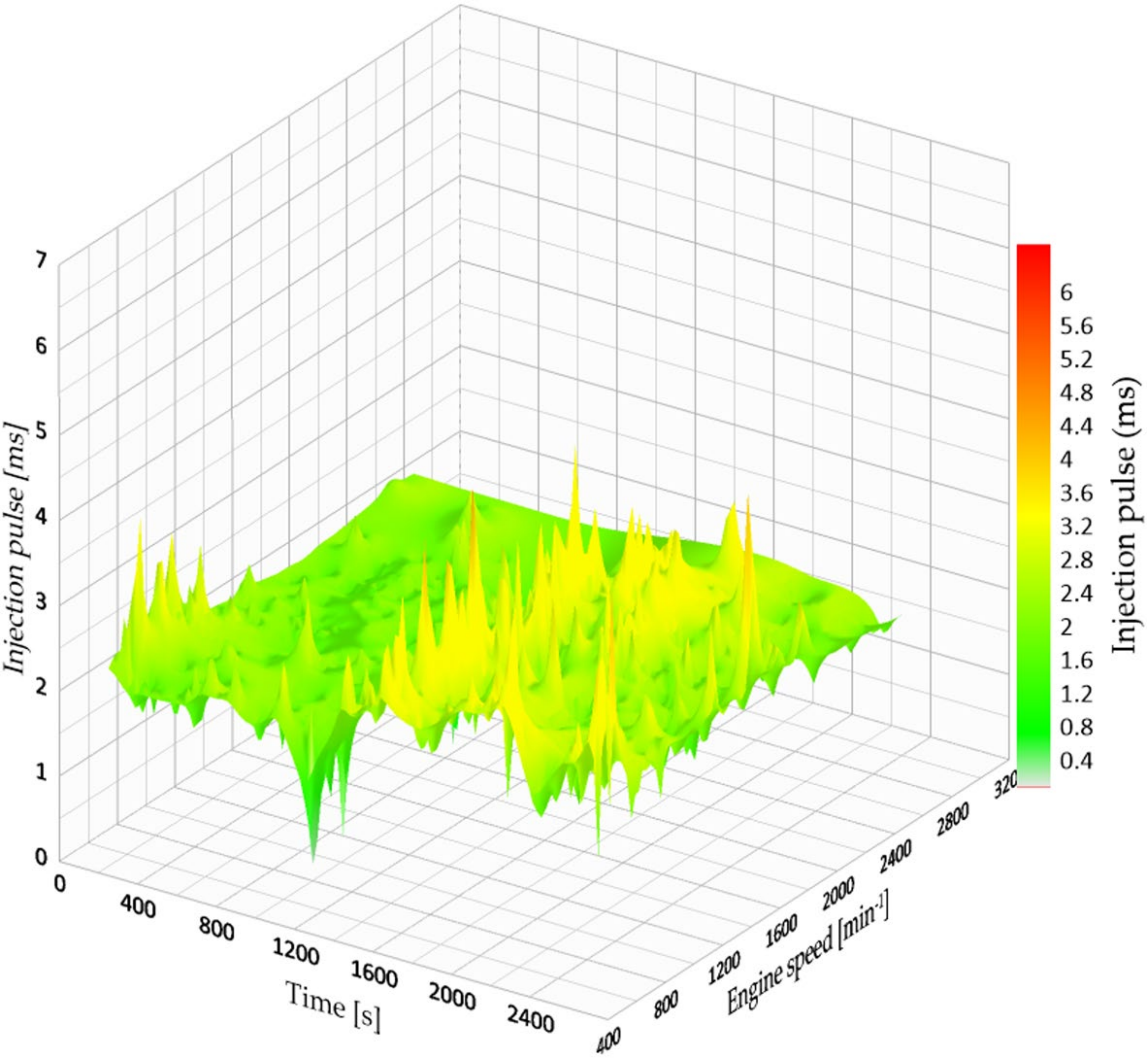
Injection map vs. engine speed at 4 bar.

At 4.0 bar, an improvement in fuel economy and engine-load behaviour was observed. The injection pulse peaks decreased to 4.63 ms, and fuel economy increased to 8.528 km/l in the evaluated route. Figure 14 indicates a more stable engine-speed profile under this setting, which is consistent with a reduction in average engine load for the tested conditions.

Figure 14 show a more consistent injection pattern characterized by diminished peaks and enhanced alignment with engine speed. The shorter pulses indicate more accurate fuel measurement and a leaner, yet still stable, combustion process.

Given an average yearly city mileage of 12,000 km, changing the tire pressure from 3.2 to 4.0 bar would lead to a reduction of about 224 L of fuel consumption each year, which corresponds to an approximate decrease of 540 kg in CO₂ emissions annually for each vehicle. When applied to a public or municipal fleet, these savings become important for both operations and the environment.

This illustrates that minor changes, like installing a fuel pressure regulator, can significantly affect the environment, particularly in urban areas where modern vehicles are limited and where traditional internal combustion engines will likely remain prevalent for the foreseeable future.

#### Fuel consumption at 4.5 bar pressure (City)

At an injection pressure of 4.5 bar, the engine demonstrated a notable enhancement in fuel efficiency, attaining one of the top performances recorded during the urban tests. The average fuel consumption attained 11.473 km/l, representing a 59.7% increase in comparison to the initial configuration (3.2 bar).

**Fig. 15:**
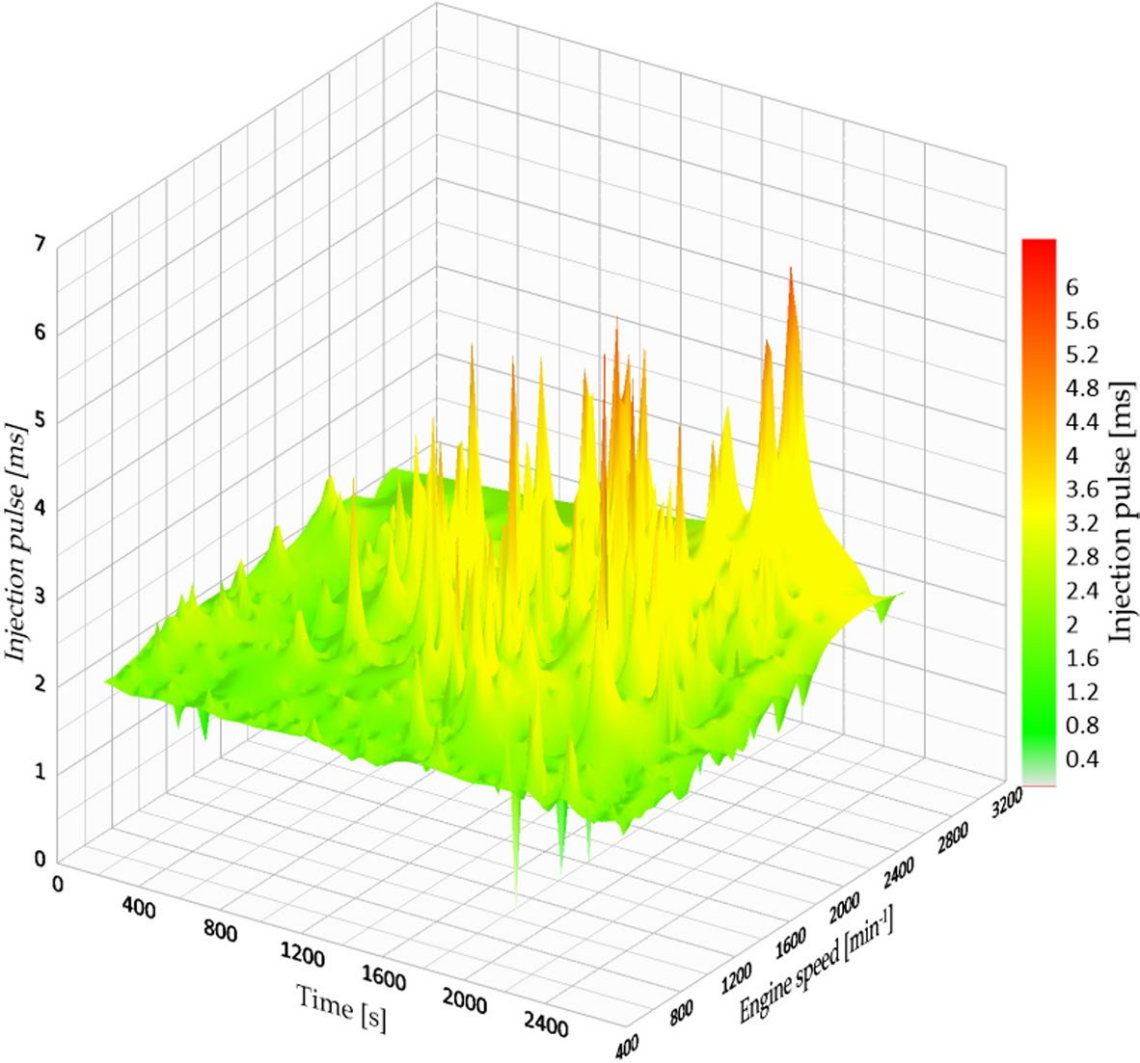
Injection map vs. engine speed at 4.5 bar.

Nevertheless, this benefit was accompanied by a compromise: while the duration of the injection pulse averaged at 5.12 milliseconds, the signal exhibited significant instability, characterized by minor peaks and fluctuations during transitional periods (for example, acceleration after coming to a complete stop). Figure 15 shows the fuel efficiency parameters at 4.5 bar.

This variation indicates that although the fuel delivery system improved its spray quality and combustion potential due to increased injection pressure, the ECU’s adaptive logic faced challenges in providing consistent fuel measurement during frequent changes in load. Such situations are prevalent in city settings, where motors need to react swiftly and often to the actions of the driver.

Nonetheless, the instability in the injection pulse indicates that, during city driving, 4.5 bar might be close to the maximum level of ideal pressure. Exceeding this level could lead to difficulties for the ECU’s compensation systems in ensuring complete control, particularly in the absence of recalibration or additional sensor integration.

This underscores a key principle of practical sustainability in engine management: the objective is not solely to reach optimal efficiency but also to ensure system stability, reliability, and adherence to emissions standards over the long term. For fleet applications, this setup might be acceptable; however, it is advisable to observe it for any long-term impacts on injector wear and mixture uniformity.

#### Fuel consumption at 5 bar pressure (City)

**Fig. 16:**
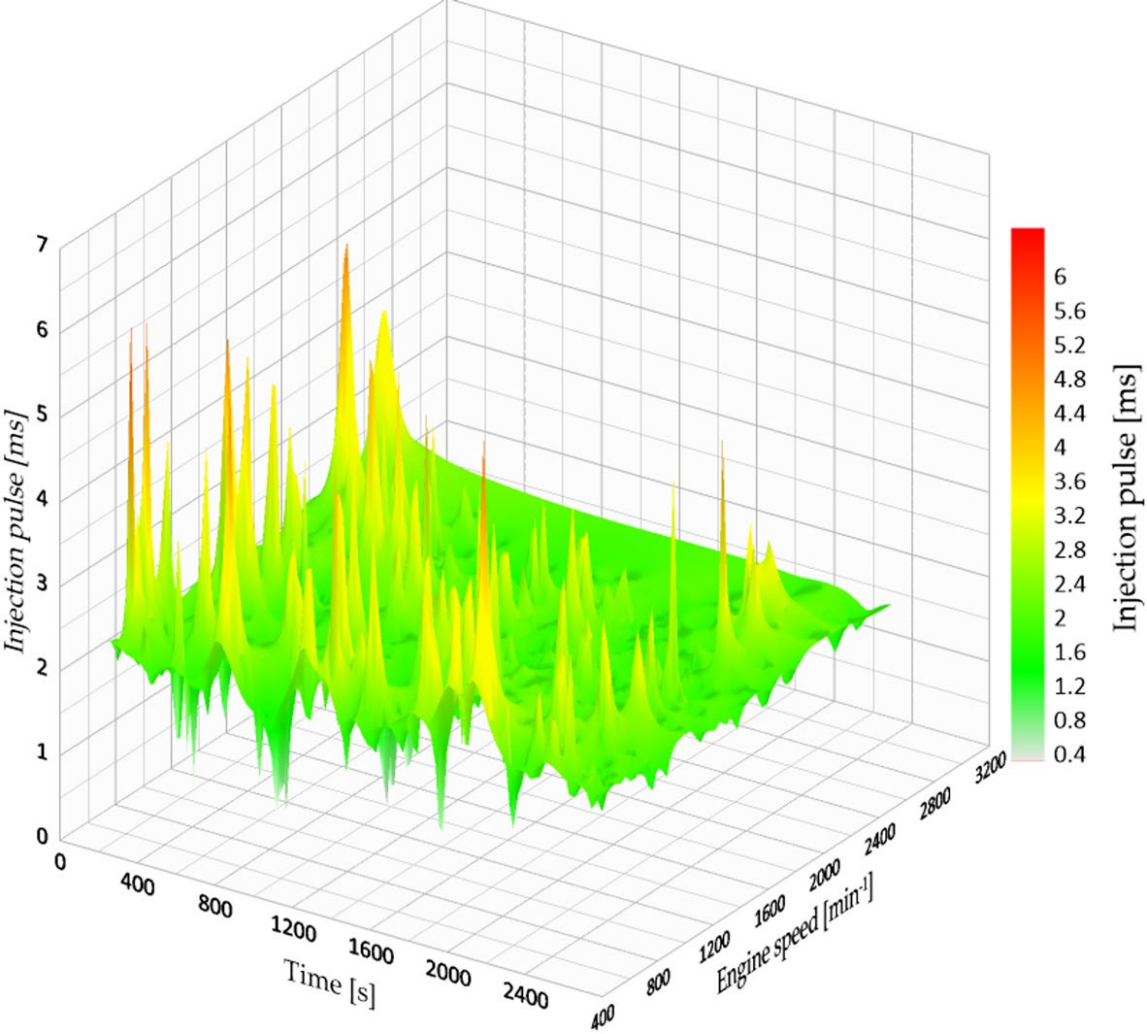
Injection map vs. engine speed at 5 bar.

Increasing the fuel injection pressure to 5.0 bar resulted in fuel consumption of 13.5 km/l under specific test conditions, representing the best performance observed in this study. The typical injection pulse measured 5.02 milliseconds, which is a bit less than the measurement at 4.5 bar. Nonetheless, like the earlier setup, the injection signal exhibited minor inconsistencies, although they were less noticeable. This indicates that even though the injector worked at higher pressure, the ECU was still able to control the mixture properly in most situations. Figure 16 shows the fuel efficiency parameters at 5 bar.

The enhancements were clear in the way the vehicle responded, decreased engine stress, and smoother operation during acceleration and braking—although there was a tendency to run lean at extremely low RPMs, which could lead to slight hesitation in specific circumstances. Even so, although the improvement in efficiency is the greatest, it is essential to thoroughly contemplate torque stability and the predictability of combustion. In severe situations, like when vehicles are heavily loaded or when traveling up steep city hills at low RPMs, this arrangement may cause problems with responsiveness. Therefore, while 5.0 bar is ideal for fuel efficiency; it may not be the most consistently stable setting for all urban driving situations.

This compromise illustrates the well-known engineering concept of diminishing returns. In this scenario, maximizing a variable lead to significant improvements in one aspect (fuel efficiency) but starts to create difficulties in other areas (drivability, adaptability). From the perspective of sustainable engineering, the optimal arrangement may be slightly below the peak—where efficiency, regulation, and emissions are harmonized.

#### Comparative summary of city test results

The urban-route tests showed a consistent increase in measured fuel economy as injection pressure was raised during the campaign. Because city driving includes repeated stops, low engine speeds and frequent load transients, the magnitude of improvement can vary notably with traffic and driver behaviour. Table 6 summarises the urban test results at each pressure level.

**Table 6:** Data resulting from tests in the city.

Fuel pressure (bar)	Injection pulse (ms)	Engine load (%)	Fuel consumption (km/l)	Test time (min)
3.2 (Original)	4.81	19.44	7.18	39.12
4.0	4.03	18.92	8.53	38.24
4.5	3.42	18.67	11.47	38.56
5.0	2.92	18.30	13.45	38.45

**Fig. 17:**
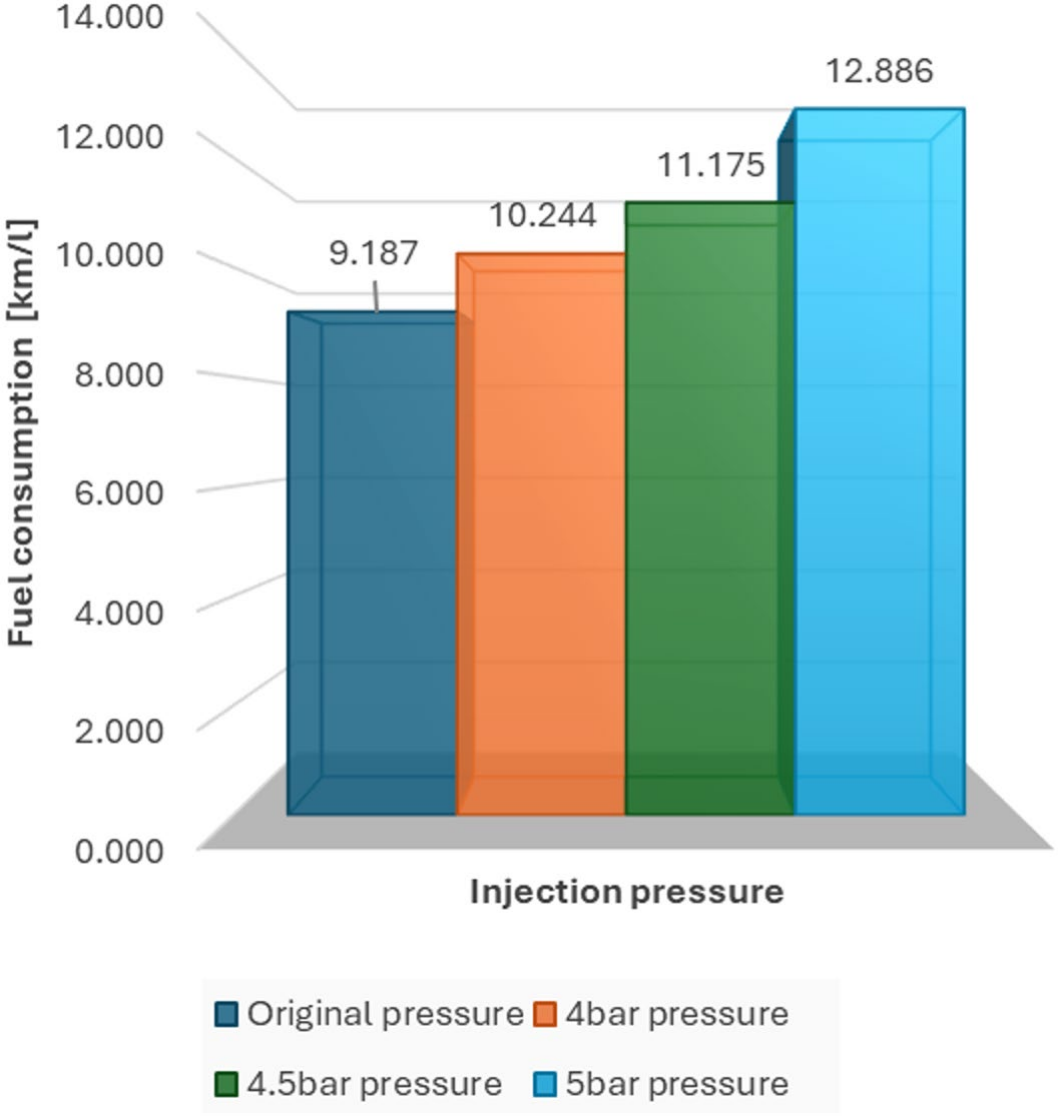
Fuel consumption in city tests.

Table 6 summarises the urban-route results. The 4.0 bar setting produced the lowest average engine load (19.88%) while maintaining engine speed within the normal operating range, indicating stable drivability in stop-and-go traffic during the test period. Under the same route, 5.0 bar produced the highest measured fuel economy (13.45 km/l versus 7.18 km/l at 3.2 bar; +87.3%). Given the inherent variability of real-road testing (traffic, driving style and route micro-conditions), these values should be interpreted as outcomes observed during the specific test campaign rather than guaranteed improvements for all vehicles or cities. Figure 17 shows the corresponding fuel-economy results for the urban tests.

**Fig. 18:**
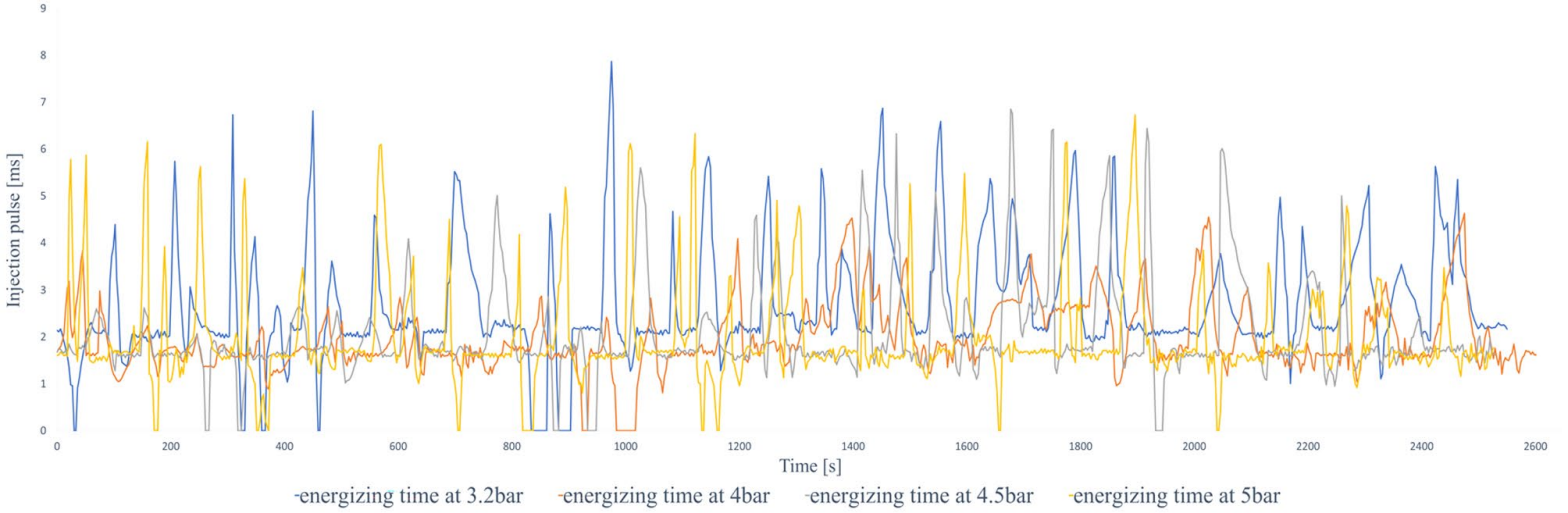
Injection pulse diagram on city routes.

**Fig. 19:**
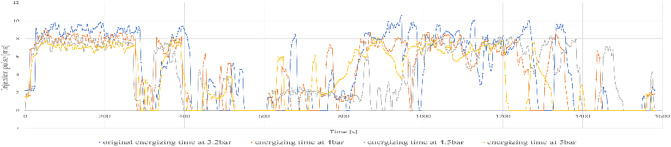
Injection pulse diagram on city routes.

Likewise, the injection diagram in Figs. 18 and 19 shows a notorious difference in the injection pulse, which has remained relatively stable.

The experimental results demonstrate substantial sustainability improvements through fuel pressure optimization. At the optimal 5.0 bar setting, urban fuel efficiency increased by 87% compared to baseline (3.2 bar) conditions, with consumption improving from 7.18 km/l to 13.45 km/l, in addition, although these results are specific to the test conditions and may not apply to all vehicles. This enhancement directly translates to proportional CO₂ emission reductions, decreasing from 320 g/km to 171 g/km based on standard gasoline emission factors (2.3 kg CO₂ per litter).

Projected annual benefits per vehicle (12,000 urban km operation).

- Fuel savings: 580 L.
- Emissions reduction: 1,330 kg CO₂.
- Economic savings: Approximately USD 580.

These findings confirm that fuel system optimization represents a cost-effective intervention for urban fleets, particularly in high-altitude environments like Quito (2,850 m AMSL) where reduced atmospheric pressure inherently compromises combustion efficiency. The methodology’s simplicity (requiring only a pressure regulator modification while maintaining original emissions controls) makes it particularly suitable for:

- Fleet operators seeking immediate efficiency gains.
- Municipalities targeting transportation emissions reductions.
- Developing regions requiring affordable sustainability solutions.

The 45% reduction in specific emissions (g CO₂/km) achieved through this mechanical intervention rivals the benefits of more complex (and costly) alternatives like hybrid conversions, while preserving vehicle warranties and requiring minimal technical expertise for implementation. This positions fuel pressure optimization as a pragmatic transitional strategy in the global shift toward sustainable mobility, especially valuable in markets where advanced electrification remains economically challenging.

#### Relationship between fuel injection pressure and engine load comportment

Further analyses were conducted to determine the relationship between fuel injection pressure and engine load under real driving conditions, thereby supplementing the investigations into fuel consumption and injection pulse. While fuel consumption values are a direct indicator of efficiency, engine load—an internal variable that integrates speed, air and fuel requirements, and ECU compensation strategies—provides a more complete picture of system behaviour. Understanding how this load changes with changes in injection pressure is key to explaining the observed increase in combustion stability and efficiency, particularly in urban and high-altitude environments.

**Fig. 20:**
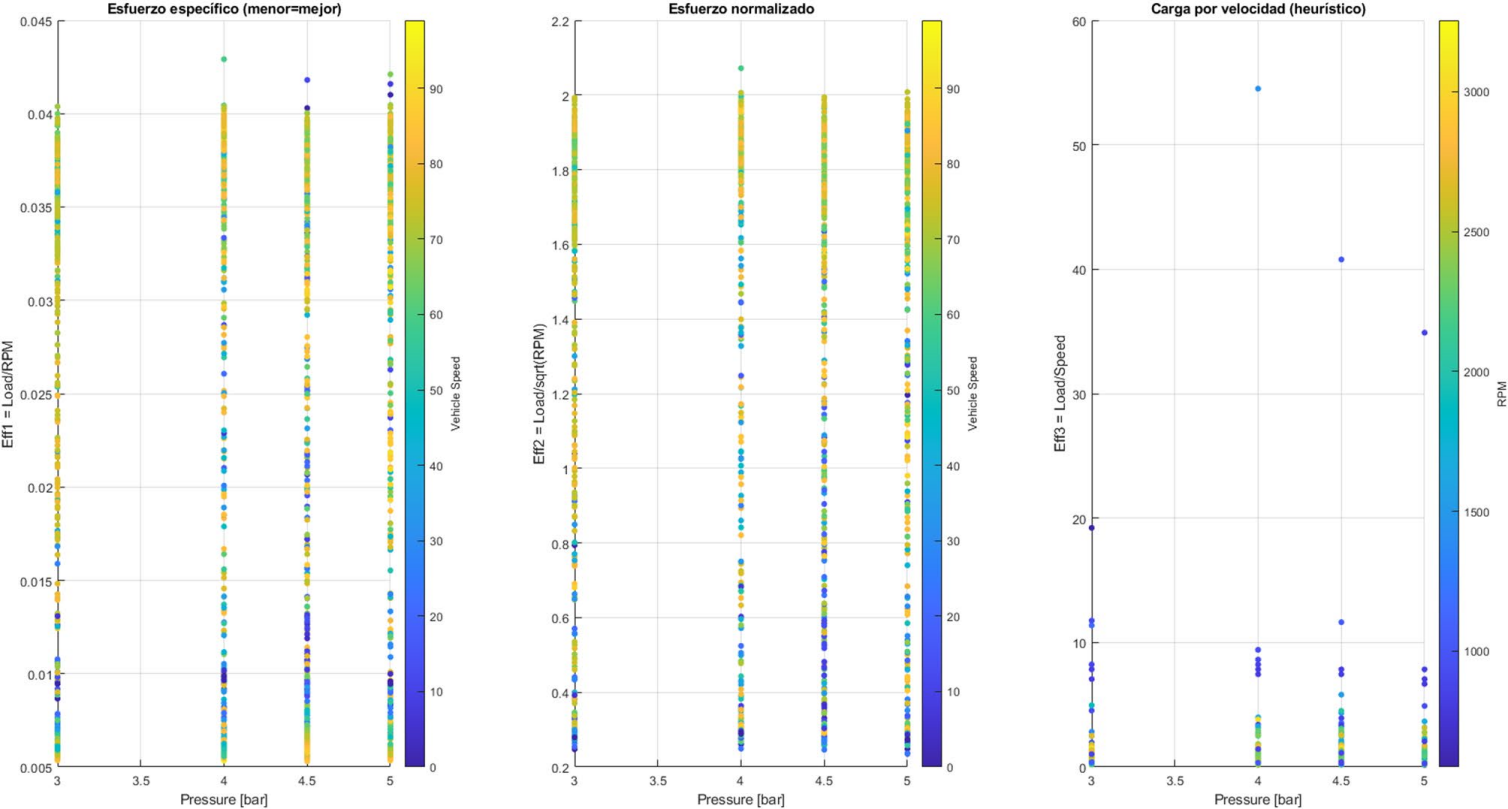
Force vs. pressure.

Figure 20 shows three performance metrics as a function of injection pressure (3.5 to 5 bar):

- Specific power (Eff1 = load / RPM) Lower values indicate better performance. It can be observed that at low or medium pressures (≈ 3.0–3.5 bar), the effort is minimal, indicating higher efficiency. At higher pressures, the effort required increases, resulting in greater friction and higher energy consumption.
- Normalized effort (Eff2 = load / √speed) This indicator partially eliminates the influence of engine speed. The increase is more pronounced at high pressures (> 4.5 bar), reflecting greater operational instability.
- Load per speed (Eff3 = load / speed) This indicator shows heuristic behaviour: the force required is greater at low speeds (city traffic) and decreases at high speeds when driving at a steady pace, which indicates more efficient energy use at constant speeds.

**Fig. 21:**
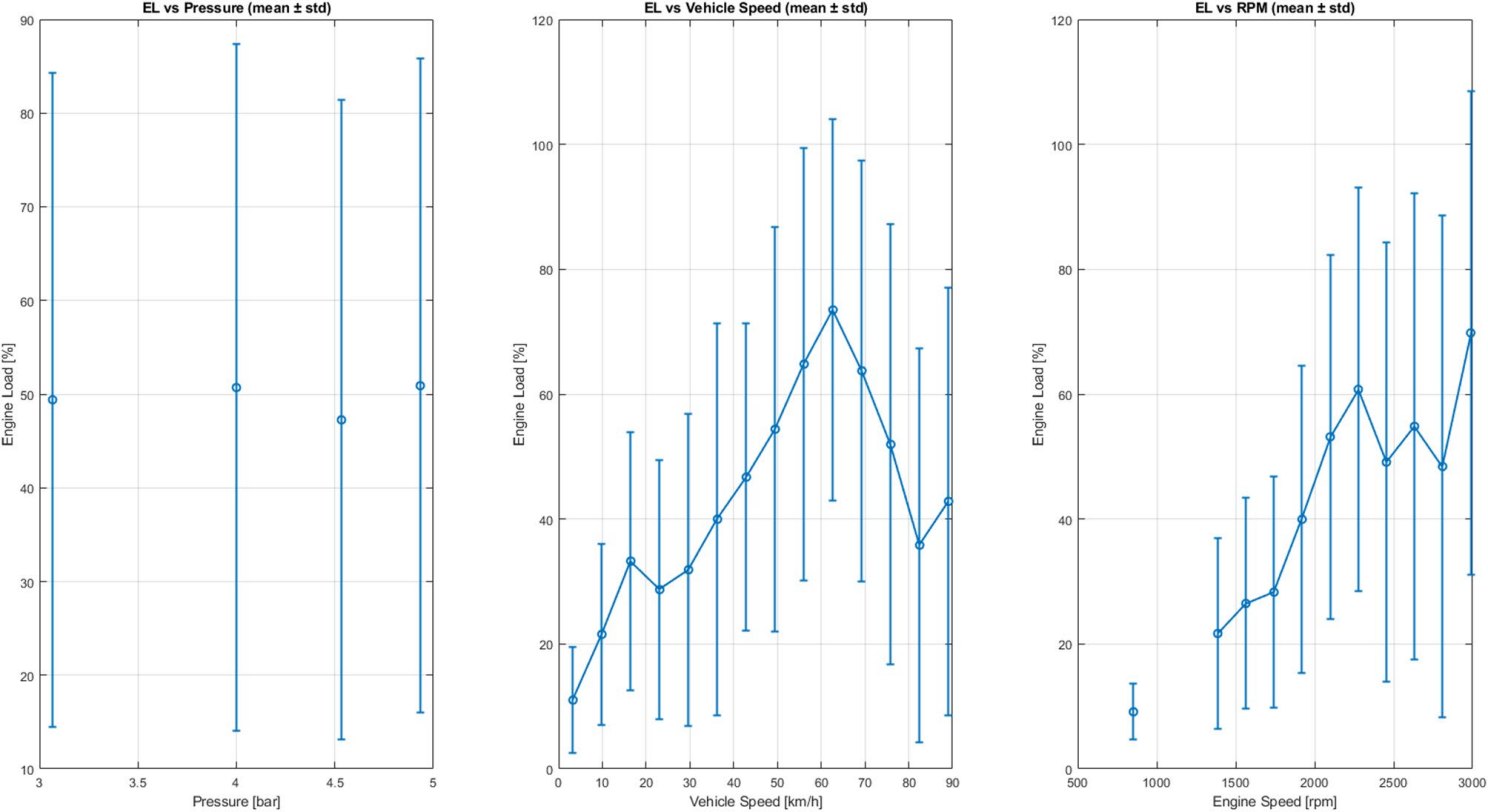
Average standard engine load behaviour.

Figure 21 shows that injection pressure, considered in isolation, does not significantly determine the power required by the engine. The average load remains close to 50% throughout the range analysed (3 to 5 bar), although considerable dispersion in the data is observed. On the contrary, power is strongly influenced by vehicle speed and engine speed, reaching its maximum values at medium speeds (approximately 60–70 km/h) and 2200–2600 RPM. These points correspond to situations where greater torque is required, and the combustion chamber achieves better filling, optimizing the air-fuel mixture. The variability observed reflects typical driving patterns in real-world scenarios, where transitions between acceleration, deceleration, and partial load are frequent.

Themselves, the results confirm that engine power is a multifactorial variable, determined mainly by revolutions and instantaneous demand, while injection pressure acts as a secondary regulator, contributing to combustion stability and overall system efficiency.

**Fig. 22:**
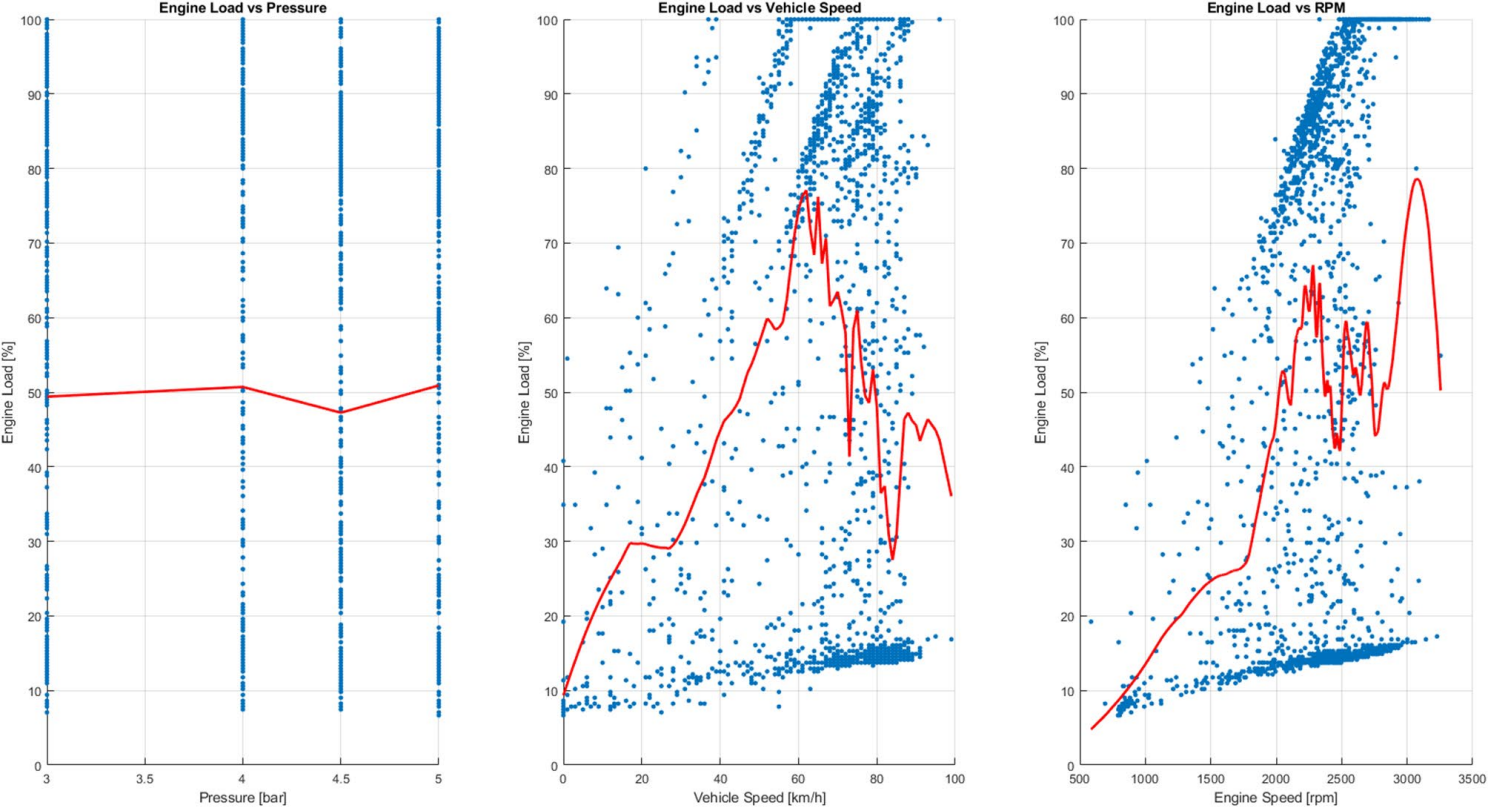
Comparison between the trend and dispersion of the motor load.

Figure 22 shows that the relationship between injection pressure and engine load remains practically constant: the average hovers around 50% across the entire analysed range (3 to 5 bar), despite the observed dispersion. This confirms that injection pressure does not directly control engine load, as the electronic management system compensates by adjusting other parameters.

Conversely, engine load manifests a strong dependence on vehicle speed and engine speed (RPM). As can be seen in the centre and right-hand graphs, the load increases progressively and reaches its maximum at medium speeds (60–70 km/h) and between 2200 and 2600 RPM. These zones correspond to the times when higher torque is required and the combustion chamber achieves more efficient filling, optimizing the air-fuel mixture.

The variability observed in these zones reflects the coexistence of different driving modes in real-world scenarios, such as acceleration, deceleration, and partial loads. In summary, the results confirm that engine load is a multifactorial variable, determined primarily by vehicle speed and instantaneous demand. Injection pressure acts as a secondary regulator, contributing to combustion stability and overall performance, but is not the dominant factor.

#### Relationship between fuel injection pressure and emissions

Exhaust gas analysis reveals a direct relationship between engine load, operating speed, and combustion efficiency. When the engine operates under high demand or with sudden variations, CO and HC emissions increase due to excessively rich fuel mixtures and uneven fuel distribution. In contrast, stable operating speeds and moderate loads—especially with low or medium injection pressures—significantly reduce these emissions, indicating more uniform combustion and more precise electronic control of the air-fuel mixture.

In contrast, a stable regime and moderate load—especially with low or medium injection pressures—significantly reduce these emissions, indicating more uniform combustion and more precise electronic control of the air-fuel mixture.

A key finding is that a stable oxygen sensor signal and less engine load fluctuation result in more consistent emissions. This confirms that proper injection pressure adjustment and optimal load management contribute to improved fuel efficiency and compliance with current emissions regulations.

**Fig. 23:**
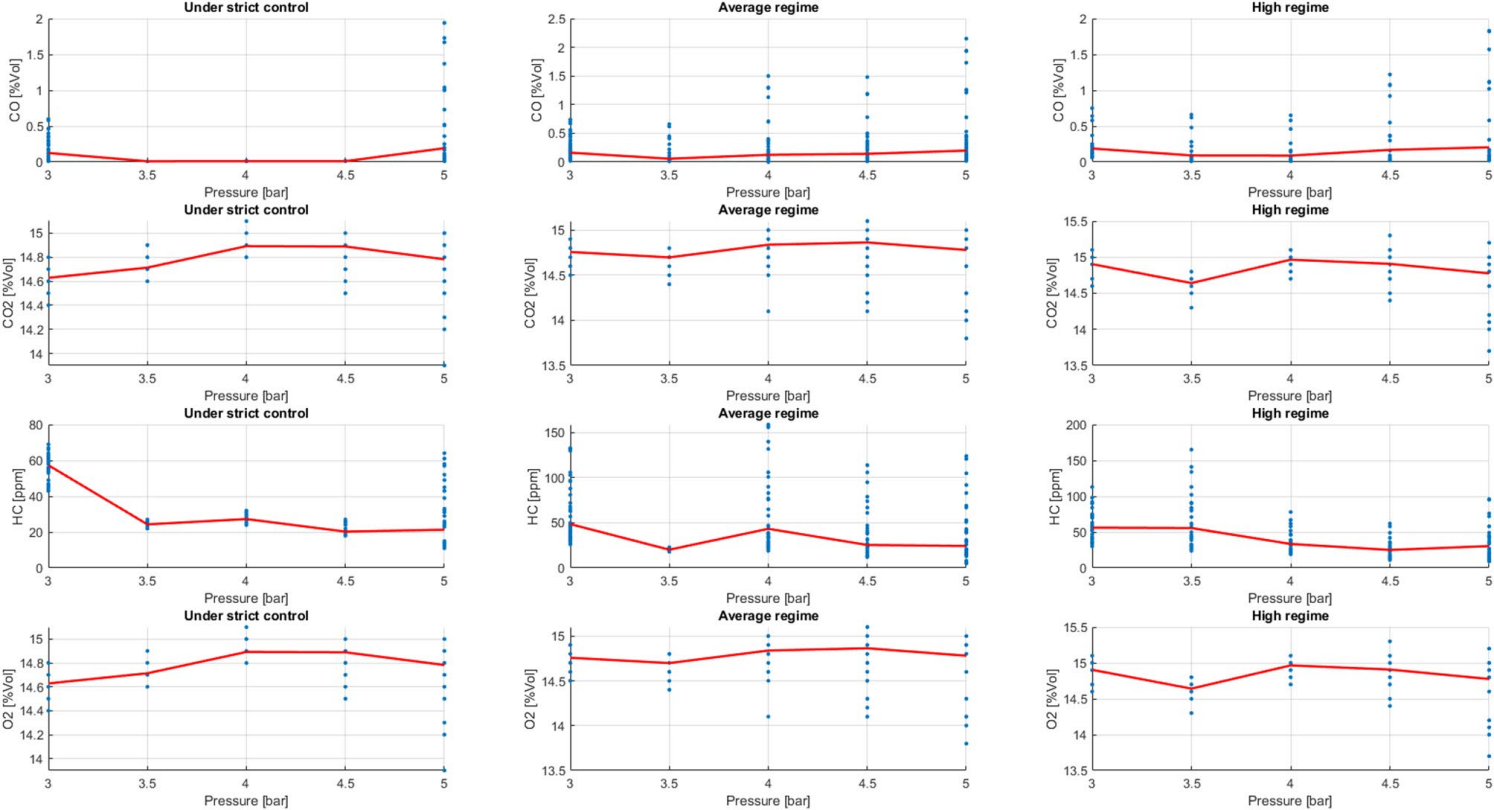
Emissions behaviour.

Figure 23 shows the typical behaviour of CO, HC, CO₂, and O₂ emissions as a function of injection pressure. CO and HC emissions decrease significantly in the medium pressure range (approximately 3.5 to 4.5 bar), indicating more complete and stable combustion. However, when the pressure increases excessively, these pollutants rise again. On the other hand, CO₂ values are slightly higher in the optimal range, consistent with more efficient combustion. The O₂ concentration remains relatively constant, indicating good control of the air-fuel mixture. In summary, the results suggest the existence of an ideal injection pressure range that minimizes harmful emissions without compromising engine performance, thus promoting both efficiency and system durability.

**Fig. 24:**
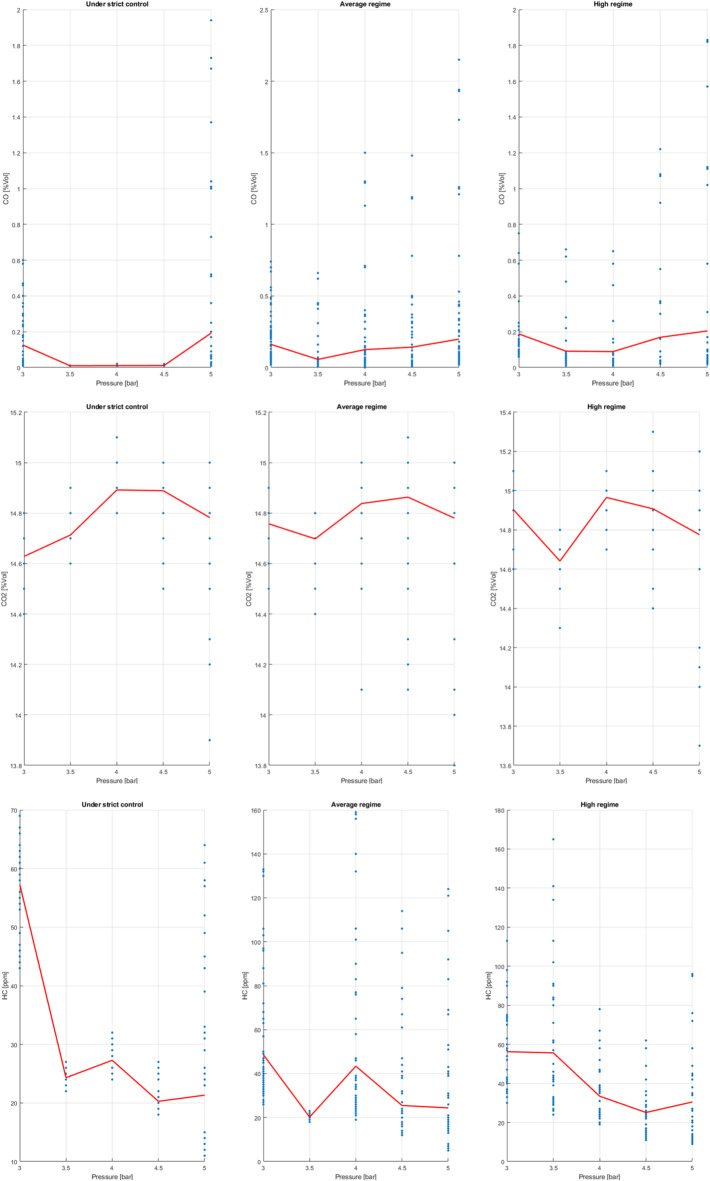
Emissions respect to pressure.

Figure 24 shows that the effect of injection pressure on emissions depends largely on the engine operating conditions. When the engine is operating at low speeds, a significant decrease in HC and CO emissions is observed with a slight increase in pressure, indicating better fuel atomization and more stable combustion, especially under low load conditions. At medium speeds, emissions reach their lowest values at intermediate pressures, which is associated with a more homogeneous air-fuel mixture and more efficient control of the electronic system. This behaviour reflects an optimal operating point where combustion is more complete. At high speeds, the general trend remains: HC and CO emissions are lower at moderate pressures. However, the dispersion of the data increases, suggesting that rapid variations in engine speed and load have a significant influence on combustion stability.

### Discussion

The findings from both urban and road tests show that changing the fuel injection pressure in a standard spark-ignition engine can lead to notable enhancements in fuel efficiency, engine performance, and environmental impact. These findings are especially important for areas that have cities at high altitudes, older vehicles, and restricted options for electric transportation. In these regions, such measures can serve as interim solutions for promoting sustainability.

#### Performance analysis

In both testing environments, raising the fuel pressure led to a steady decrease in the injection pulse width, enabling shorter fuel delivery intervals and improved atomization. This results in more efficient burning and reduced fuel use. The workload of the engine significantly decreased at higher pressures, particularly during road tests, where the demand for torque increases because of changes in elevation and prolonged speeds.

In urban evaluations, the highest efficiency was noted at 5.0 bar, achieving a fuel consumption of 13.45 km/l, in contrast to only 7.18 km/l under factory conditions. On the highway, a pressure of 5.0 bar achieved the highest fuel efficiency: 12.89 km/l, compared to the initial 9.19 km/l. Nonetheless, at 5.0 bar, mild low-RPM torque shortfalls were observed, especially in stop-and-go scenarios, indicating that although peak efficiency is attained, the balance of the system and the ease of driving may experience a slight decline without the recalibration of the ECU.

#### Optimal configuration trade-offs

Although 5.0 bar showed the best fuel efficiency, 4.5 bar was identified as the best pressure level when considering all factors—fuel savings, stability of the injection pulse, torque response, and reliability of the system.

- At a pressure of 4.5 bar, the rate of fuel consumption was:
  - 11.17 km per litre on the road (a 21.6% increase compared to 3.2 bar).
  - 11.47 km per litre in urban areas (an enhancement of 59.7%).
- The injection pulses were brief and consistent, which reduced fluctuations in ECU compensation.
- The performance of the engine was stable and reliable throughout both cycles.

This balance of 4.5 bar is an ideal setting for situations where daily dependability is essential, such as in logistics, delivery vehicles, or vehicles used by public services.

#### Practical and sustainable application

These results suggest that low-cost adjustments to fuel delivery may help reduce fuel demand in high-altitude urban operation, which is relevant to sustainable mobility discussions in cities. By lowering fuel consumption, the approach is aligned with resource efficiency and can contribute to lower greenhouse-gas emissions when scaled; however, this study did not include direct tailpipe pollutant measurements, and CO₂ changes should be interpreted as indicative based on fuel-consumption differences.

From an applied perspective, the method illustrates an incremental, retrofit-oriented intervention that could be evaluated alongside other demand-reduction measures in contexts where rapid electrification is constrained. Any potential deployment should be preceded by validation across a representative fleet, durability assessment, and compliance testing against the applicable emissions regulations.

#### Limitations

This work is a case study conducted on a single MPI vehicle platform and a limited set of routes in Quito (2,850 m.a.s.l.); therefore, the magnitude of the observed fuel-economy changes may not generalise to other engines, control strategies, fuels, altitudes or traffic conditions. Real-road tests are sensitive to driver behaviour, congestion, road grade and ambient conditions, and no chassis-dynamometer repetition was performed to quantify test-to-test uncertainty. The study relied on ECU/OBD signals to interpret injection behaviour, and the coupled effects of altered rail pressure and MAP-signal conditioning could not be fully disentangled. Long-term durability (injector/pump wear, catalyst impacts) and direct tailpipe emissions (CO, HC, NOx and particulate number) were not assessed; therefore, emissions benefits should be considered indicative and primarily inferred from fuel-consumption differences. Future work should address these limitations using a larger fleet, controlled test cycles and direct emissions instrumentation (e.g., PEMS) to confirm the real-world emissions impact.

## Conclusions

Under the conditions of this case study at high altitude (Quito, 2,850 m. a.s.l.), varying the fuel-rail pressure of an MPI spark-ignition engine using an external pressure regulator and a simple electronic interface was associated with measurable changes in fuel economy and injection-pulse behaviour during real-road driving.

The 5.0 bar setting produced the highest measured fuel-economy values in the tested urban and highway routes (7.18→13.45 km/l and 9.19→12.89 km/l, respectively), whereas 4.5 bar offered a more stable drivability compromise in congested low-RPM operation. These results indicate that pressure optimisation may be a practical lever for improving efficiency in specific high-altitude operating regimes without ECU recalibration, although the mechanistic causes should be confirmed with dedicated combustion and spray diagnostics.

Because the evaluation was limited to one vehicle and did not include durability testing or direct tailpipe measurements, the results should not be extrapolated to fleet-wide savings or regulatory compliance without further validation. Nevertheless, the approach illustrates an incremental pathway that could complement broader decarbonisation strategies (electrification, fuel quality improvements and traffic management) in regions where near-term constraints slow rapid transitions, but it is important to note that these findings must be confirmed in a larger range of vehicles and different driving contexts. Further studies are essential to determine the applicability of these results to other vehicle models and operating conditions.

These findings offer a pragmatic pathway for optimising existing vehicle fleets in developing regions, where immediate electrification remains impractical. The study proposes a strategy that could contribute to optimising fuel efficiency under certain conditions, although its effectiveness in other vehicles and situations requires further validation. Ultimately, this work exemplifies how targeted engineering interventions can deliver meaningful environmental benefits during the transition to cleaner transportation systems.

### Recommendations

The objective is to expand the experimental range. Tests will be conducted at pressures exceeding 5 bar and under varying load, altitude, and temperature conditions to rule out the possibility of deterioration at high pressures and establish safe operating limits and the robustness of the method under extreme conditions, ensuring its applicability in regions with significant geographical variations. In this context, the system’s durability and reliability will also be evaluated. It is essential to analyse the wear of injectors, pumps, and components at high pressures through fatigue testing and long-term tribological studies.

Characterisation of emissions and environmental studies. Future research within the project aims to include direct measurements of pollutants to compare the improvement in consumption with the actual reduction in emissions. It is also advisable to include comparisons with international standards (Euro VI, EPA) to ensure compliance with regulations.

## Supplementary Information

Below is the link to the electronic supplementary material.

Supplementary Material 1

Supplementary Material 2

Supplementary Material 3

Supplementary Material 4

Supplementary Material 5

Supplementary Material 6

Supplementary Material 7

Supplementary Material 8

Supplementary Material 9

Supplementary Material 10

Supplementary Material 11

## Data Availability

All data generated or analysed during this study are included in this published article and its supplementary information files.

## Abbreviations

SI: Spark-ignition
ICE: Internal combustion engine
MPI: Multi-point injection
ECU: Engine control unit
MAP: Manifold absolute pressure
OBD-II: On-board diagnostics II
CAN: Controller area network
PWM: Pulse-width modulation
IAT: Intake air temperature
TPS: Throttle position sensor
RDE: Real driving emissions
GVWR: Gross vehicle weight rating
SDGs: Sustainable development goals
CO: Carbon monoxide
CO_2_: Carbon dioxide
NOx: Nitrogen oxides
HP: Horsepower
RPM: Revolutions per minute
GVWR: Gross vehicle weight rating

## Symbols

p: Pressure, Pa, kPa, bar
p_atm_: Atmospheric pressure, kPa
p_man_: Manifold absolute pressure, kPa
P_inj_: Fuel injection pressure, bar
τ: Torque, N m
ω: Angular speed, rad s^−1^
n: Engine speed, min^−1^ (RPM)
$\dot{m}_{air}$ _air: Air mass flow rate, g s^−1^
$\dot{m}_{fuel}$: Fuel mass flow rate, g s^−1^
AFR: Air–Fuel Ratio, —
λ: Lambda factor, —
PW: Injection pulse width, ms
v: Vehicle speed, km h^−1^
a: Acceleration, m s^−2^
η_th_: Thermal efficiency, —
FC: Fuel consumption, km l^−1^ or l·100 km^−1^
E_CO2_: CO_2_ emissions, g km^−1^

## Greek symbols

λ: Lambda factor
τ: Torque
ω: Angular velocity
η: Efficiency

## Units

Pressure: Bar, kPa
Torque: Nm.
Speed: Km h^−1^, min^−1^
Temperature: °C
Altitude: M MASL
min^–1^: Revolutions per minute
Ms: Milliseconds
Nm: Newton meter
Km/l: Kilometre/litre
m. a. s. l.: Meters above sea level

## Standards and references

SAE J1321: The Fuel Economy Test Procedure (Type II)
SAE J1349: The Power Rating Standard (Engine Power Certification)
SAE J1979: The Diagnostic “Conversation” (OBD-II)
SAE J1939-71: The “Language” (Application Layer) network protocol
