# Supplementary material for: Effect of air and fuel injection pressure variation on torque and fuel economy in spark-ignition engines
Source: Sci Rep. 2026 Mar 4;16:11955. doi: 10.1038/s41598-026-41765-z (PMC13069035; doi:10.1038/s41598-026-41765-z)

Figure S1. ARDUINO MEGA 2560

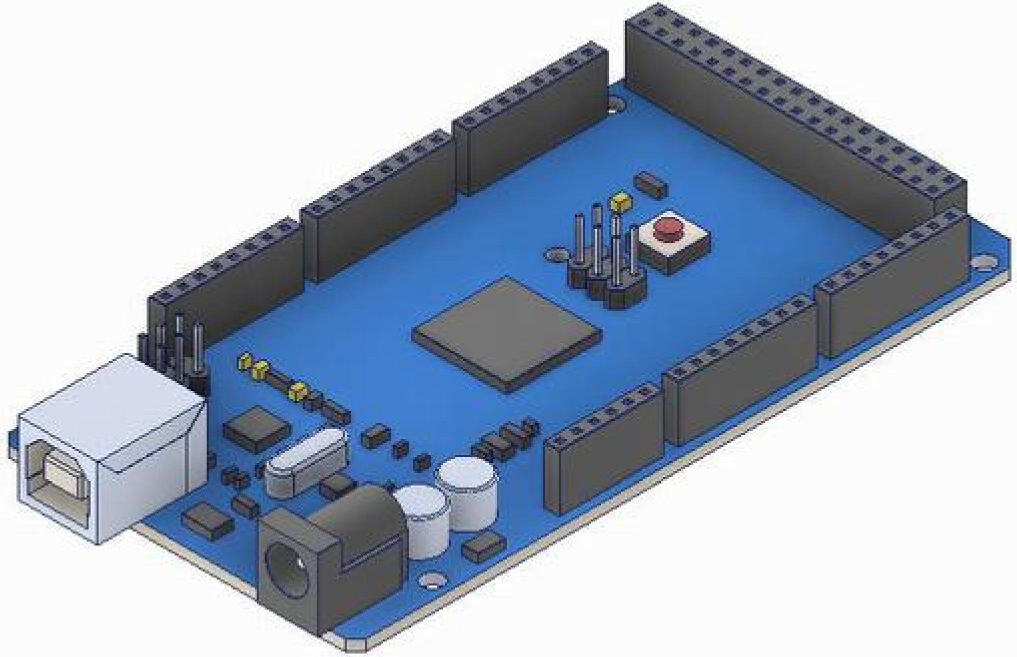

Figure S2. GRAVIMETRIC BALANCE

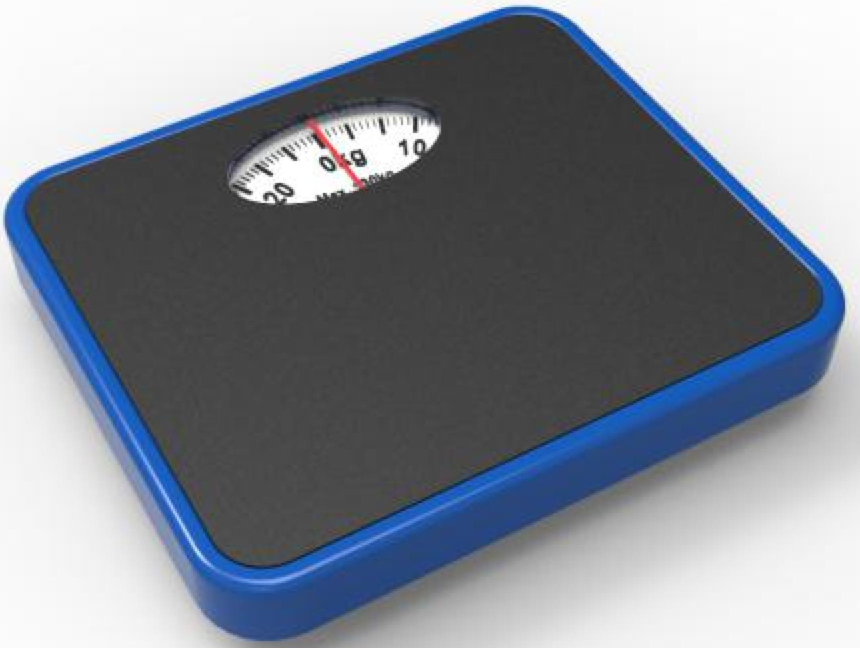

Figure S3. ELECTRONIC CONTROL UNIT

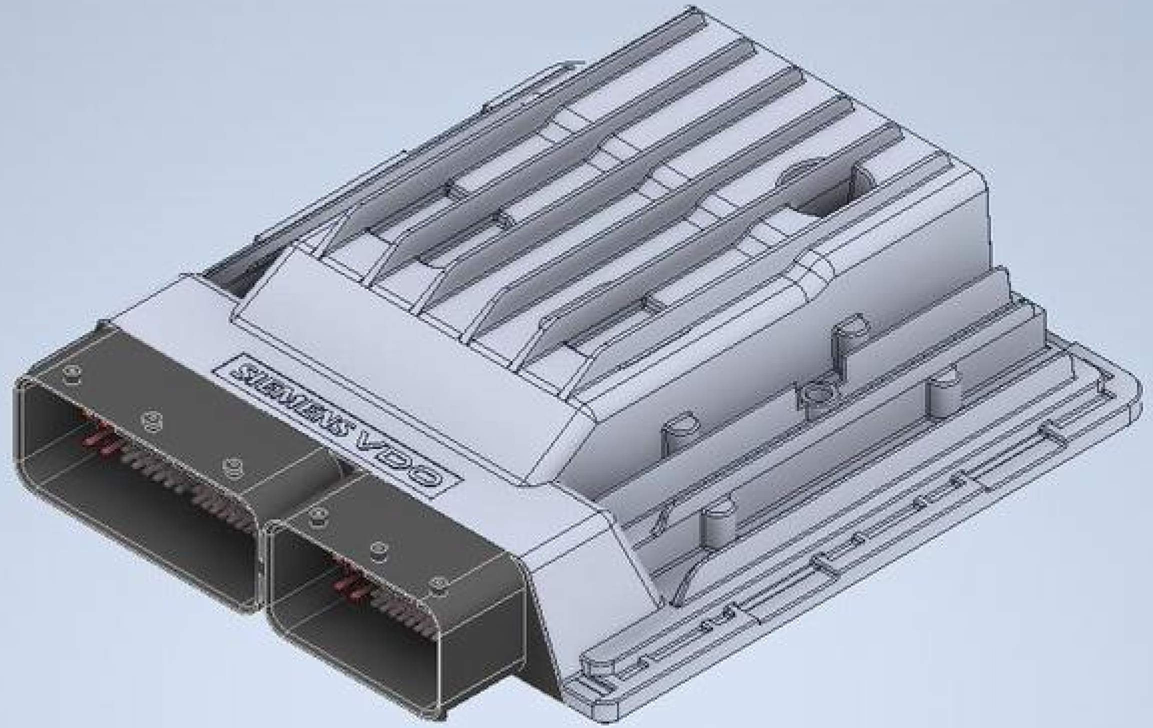

Figure S4. COMPARATIVE FUEL CONSUMPTION

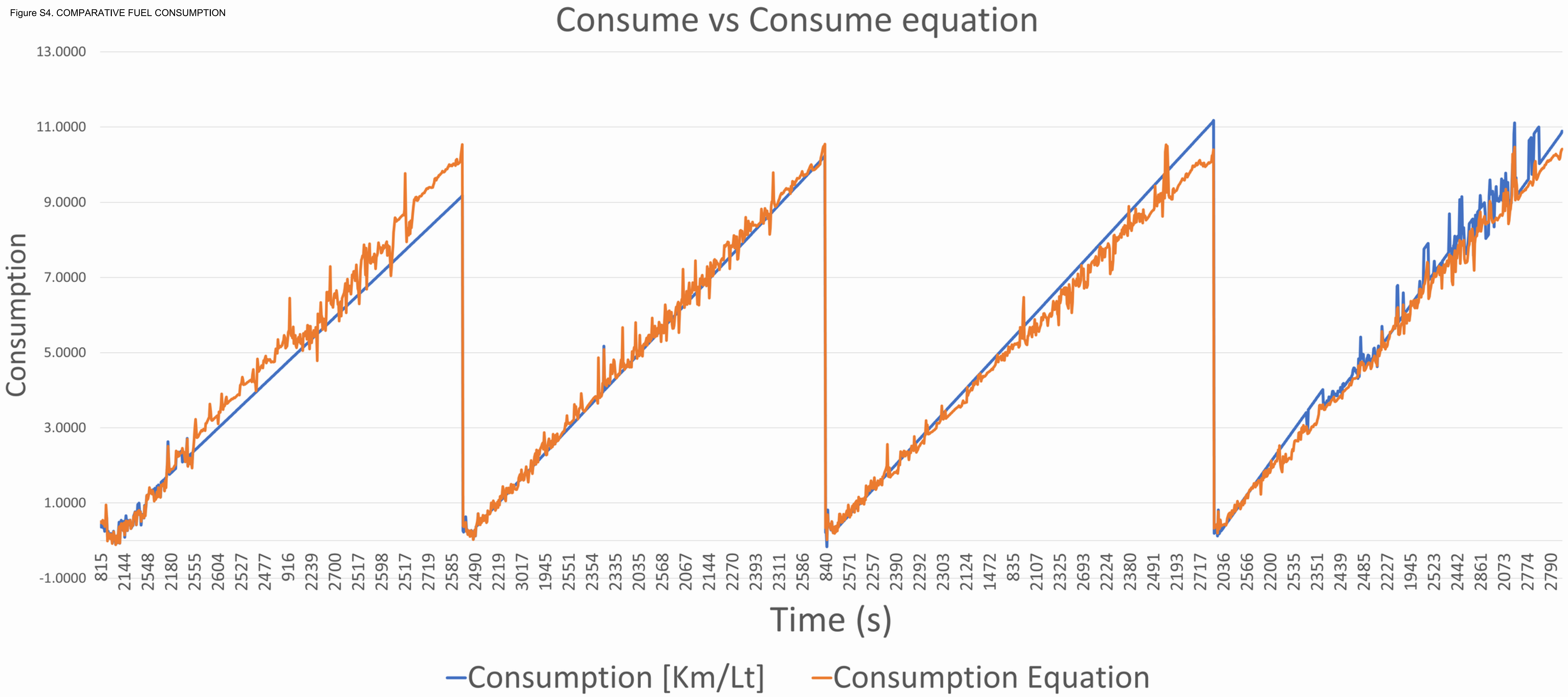

Figure S5. FUEL RAIL

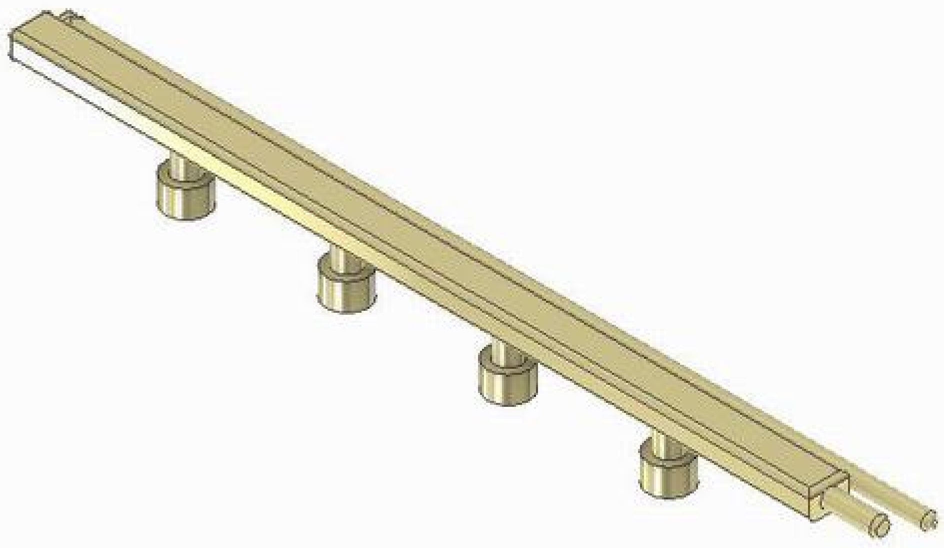

Figure S6. PICK UP VEHICLE

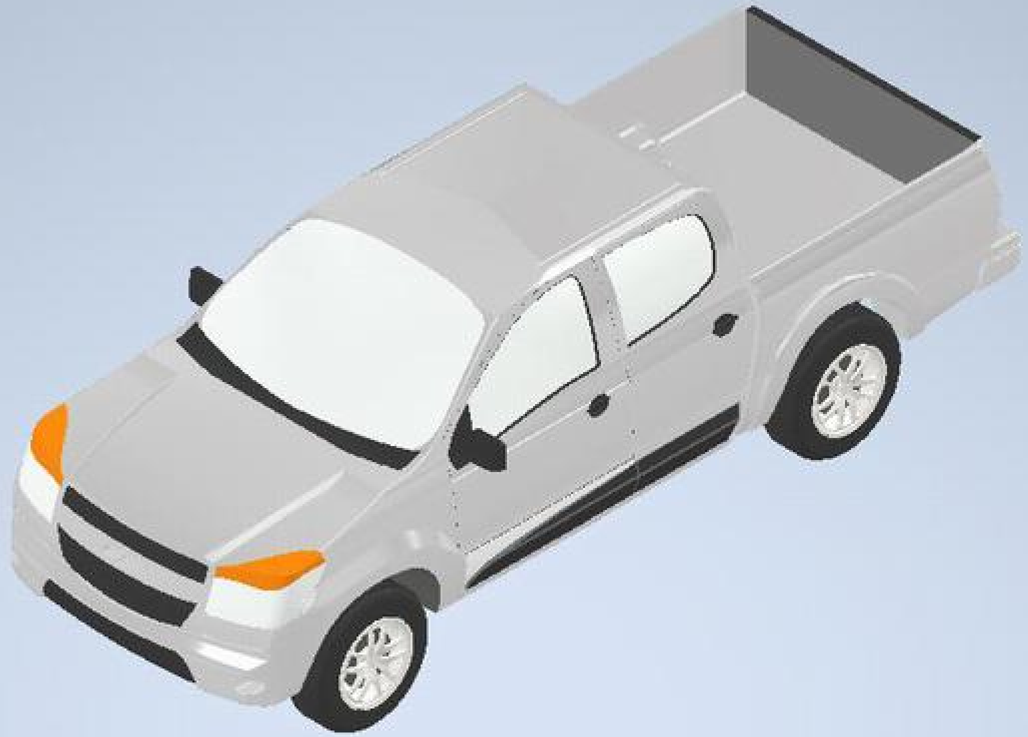

Figure S7. AUTOMOTIVE SCANNER

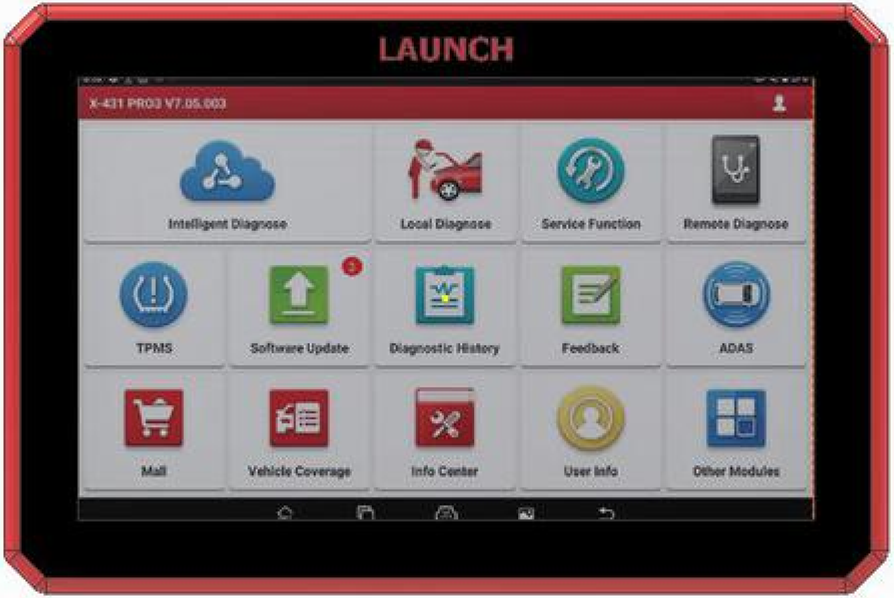

Figure S8. CONTROL SIGNAL DIAGRAME

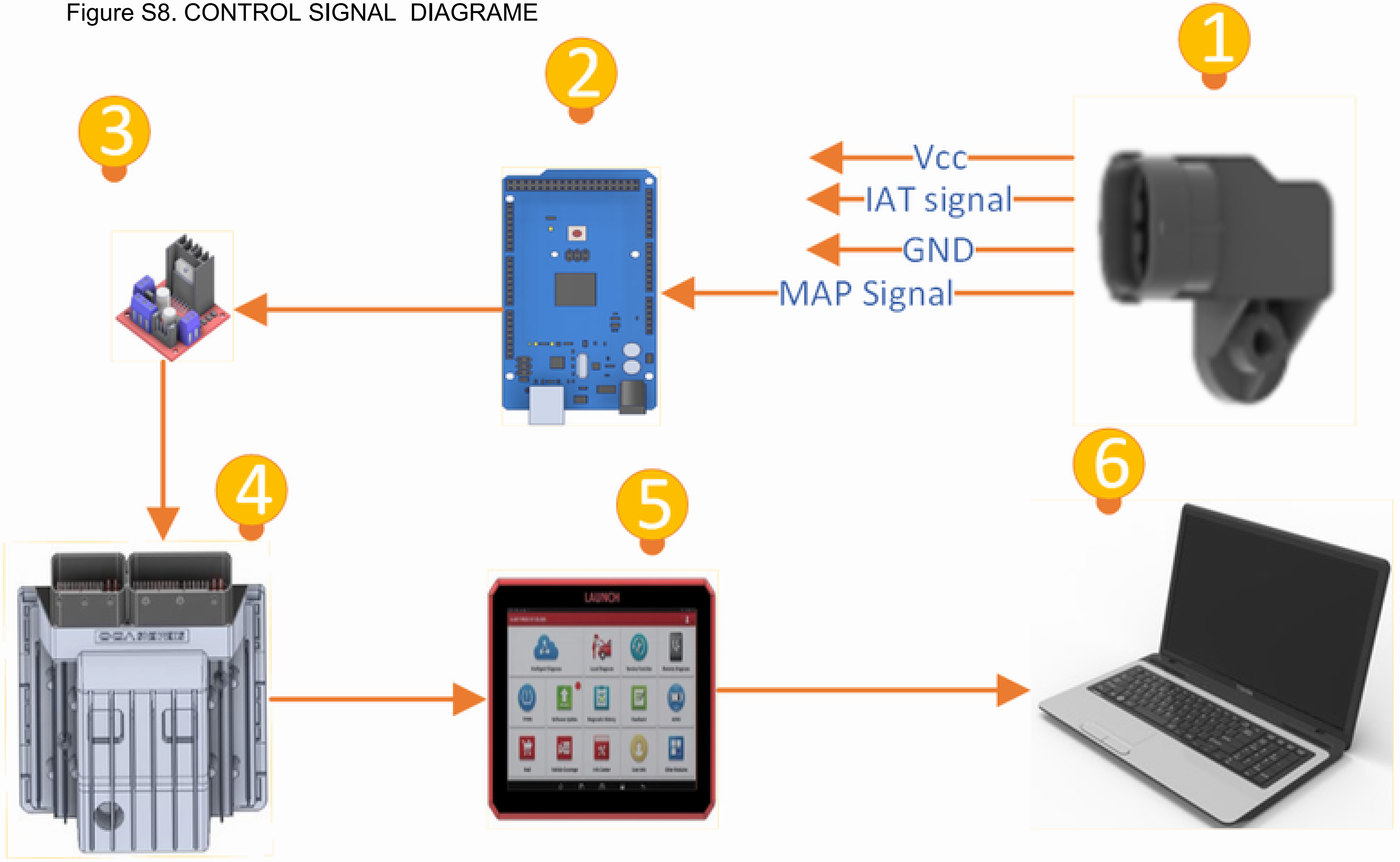

Equipment list

1) MAP Sensor.
2) Control card.
3) Signal rectifier module.
4) ECU
5) Automotive scanner
6) Pc.

Figure S9. FUEL TANK

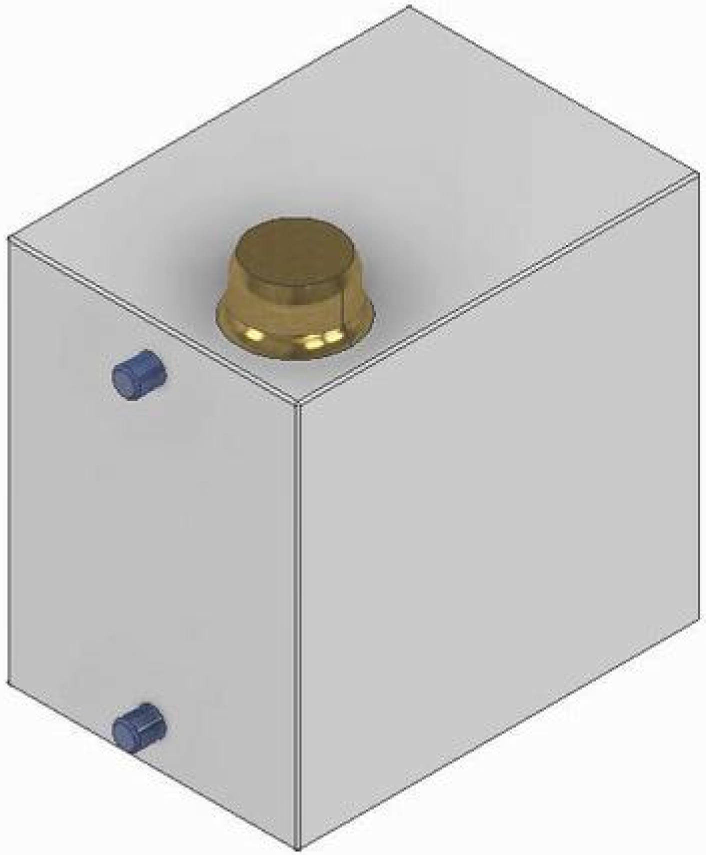

Figure S10. CO vs. pressure

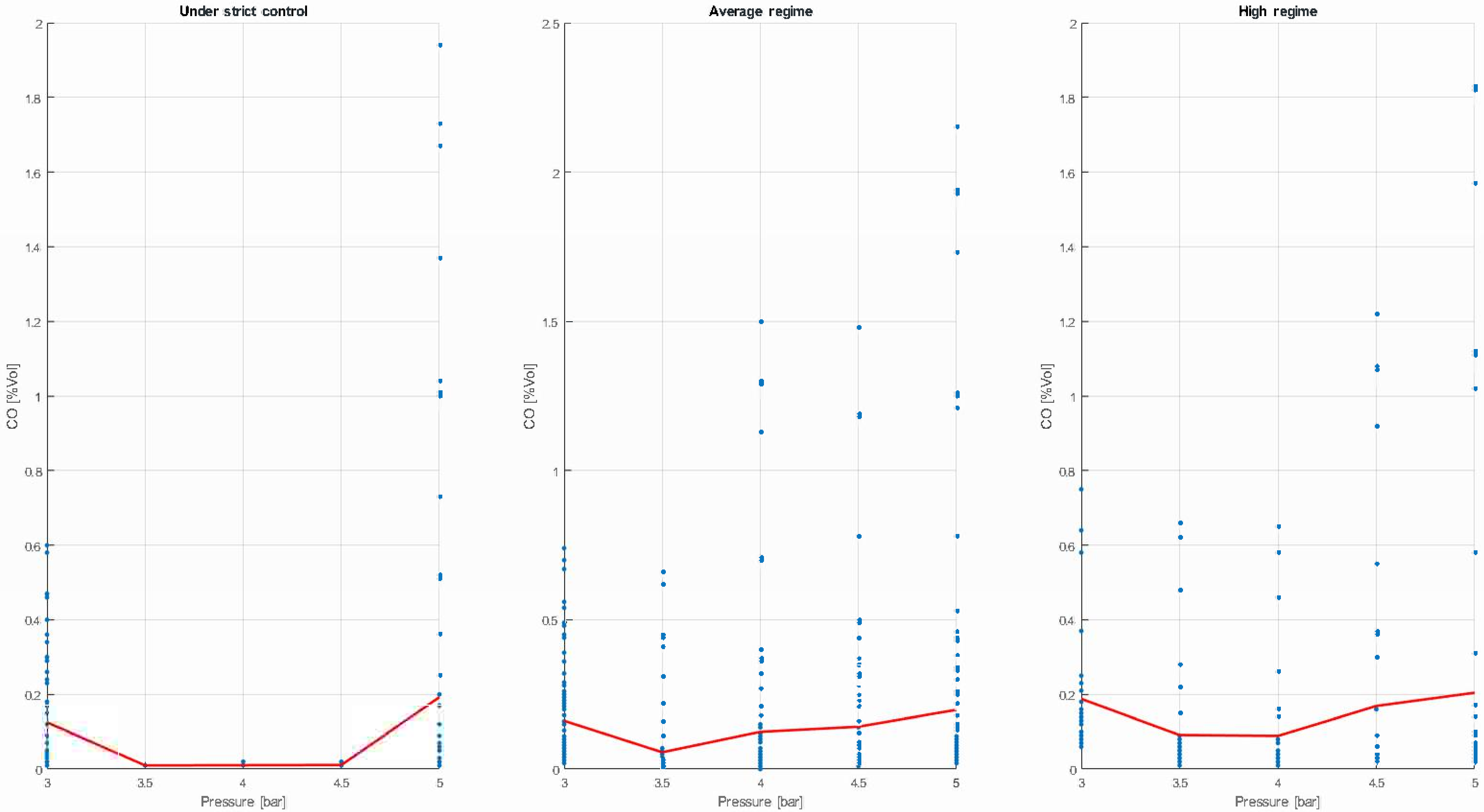

Figure S11. CO_2 vs. pressure

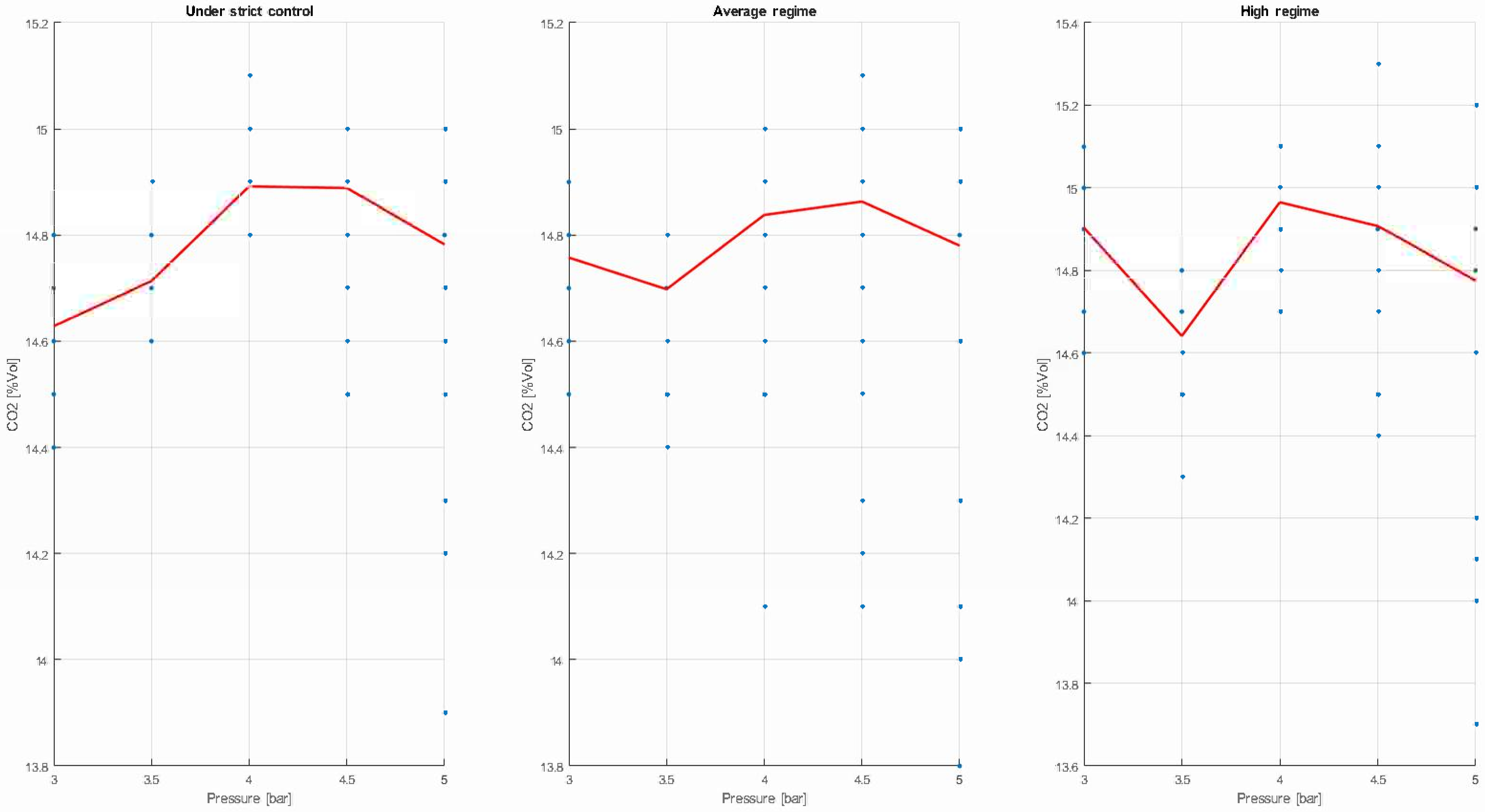

Figure S12. Comparative pressure vs. engine load

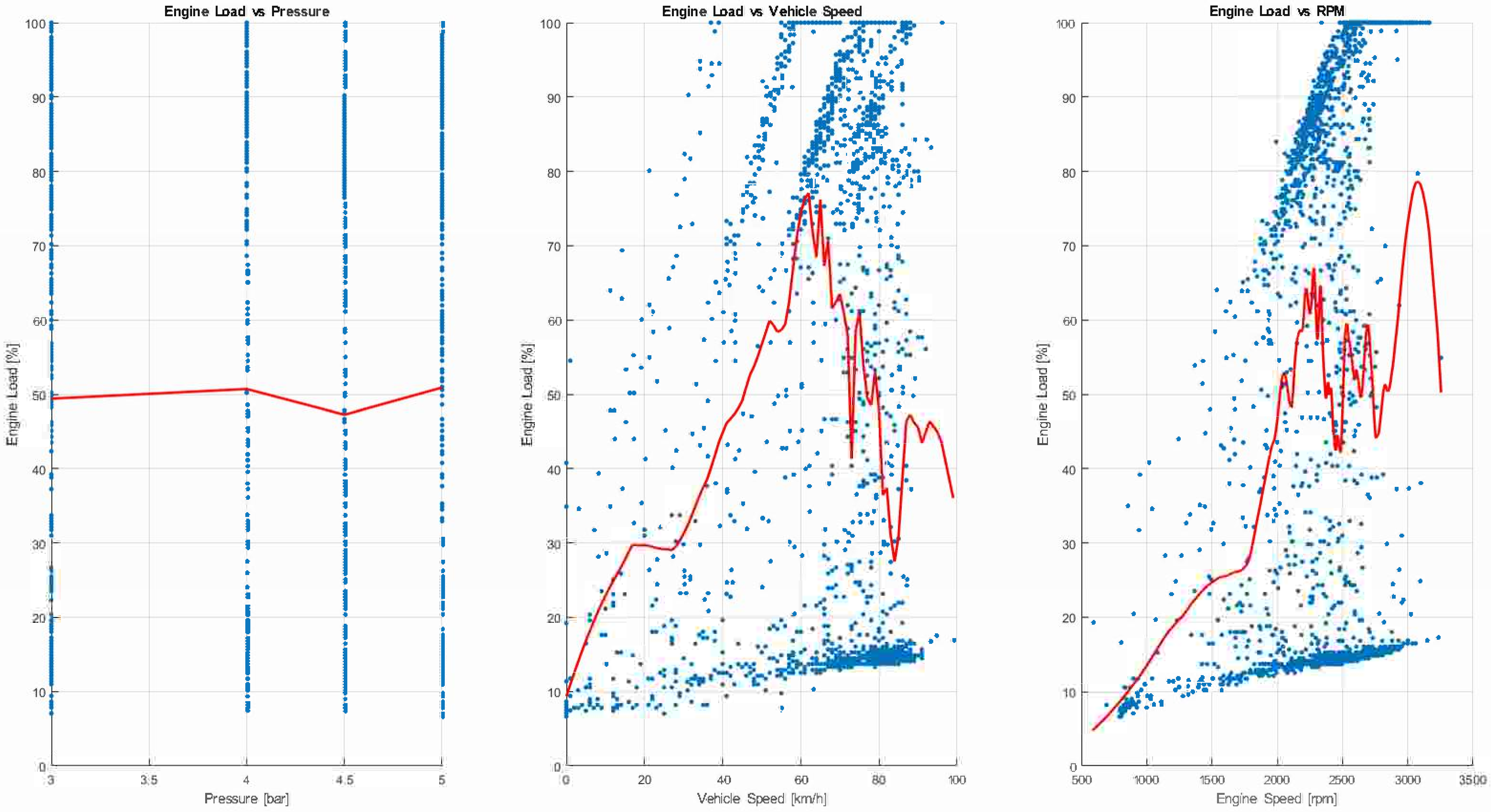

Figure S13. Total emissions

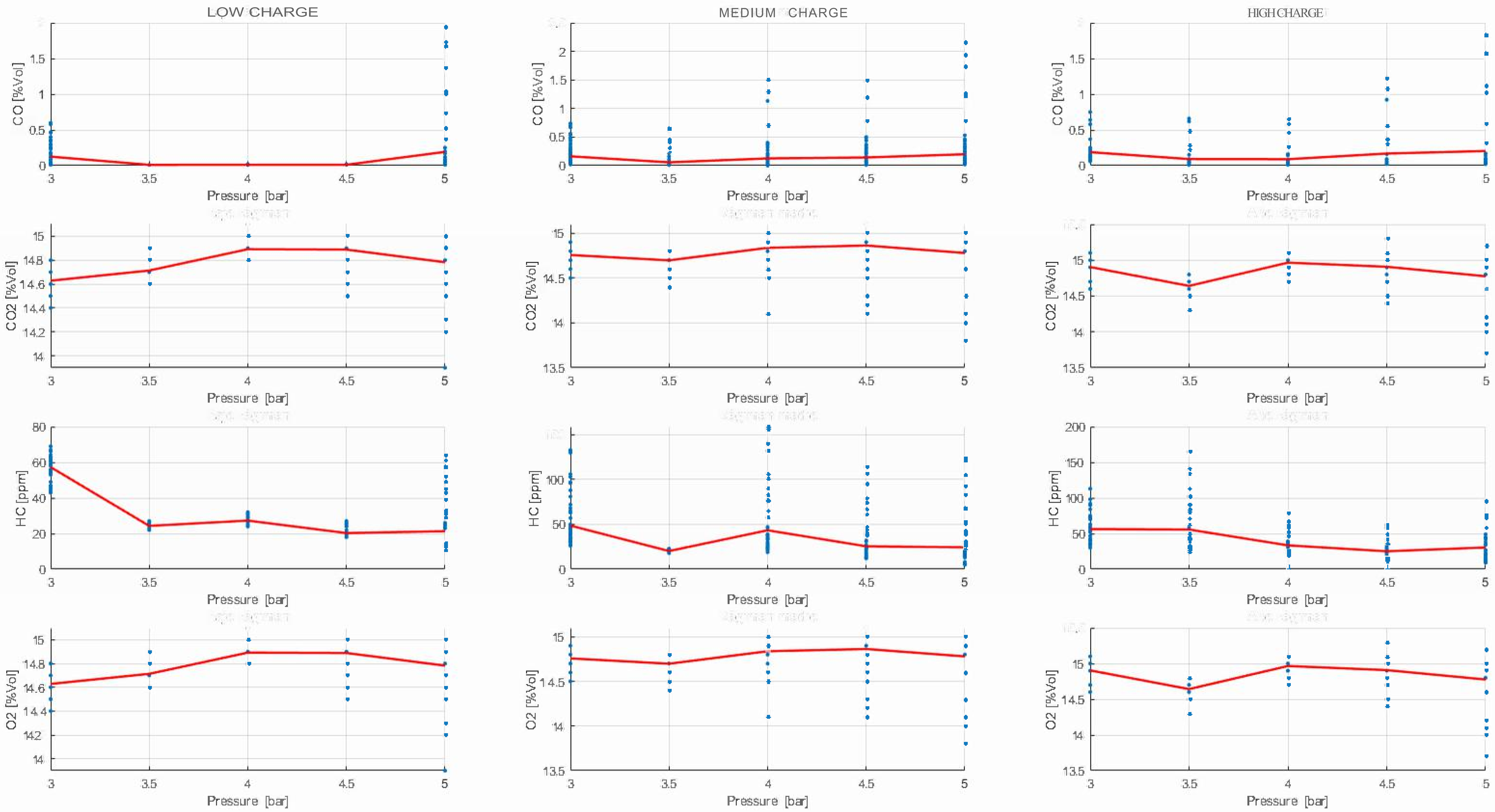

Figure S14. Emissions vs. pressure medium

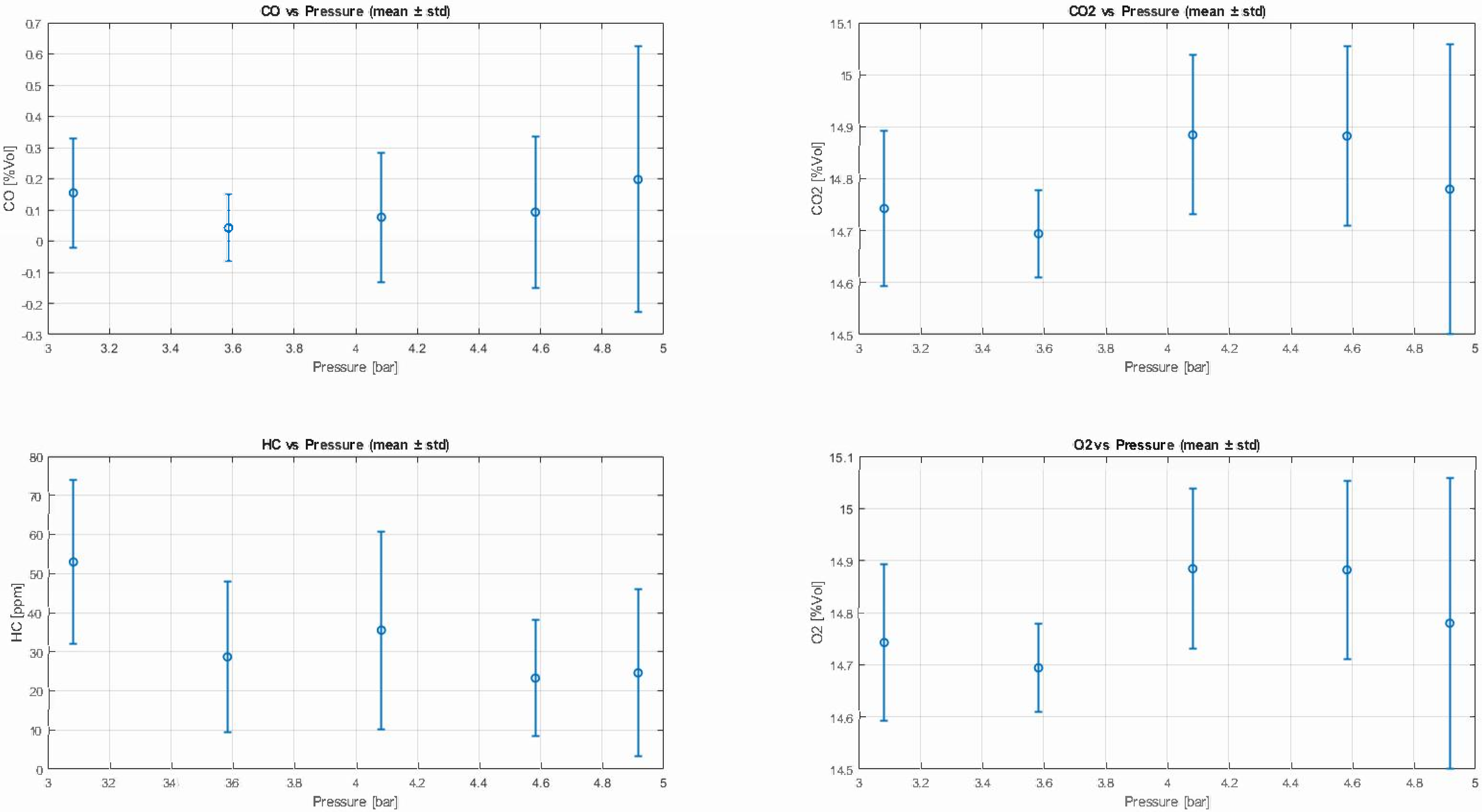

Figure S15. Emissions vs. pressure

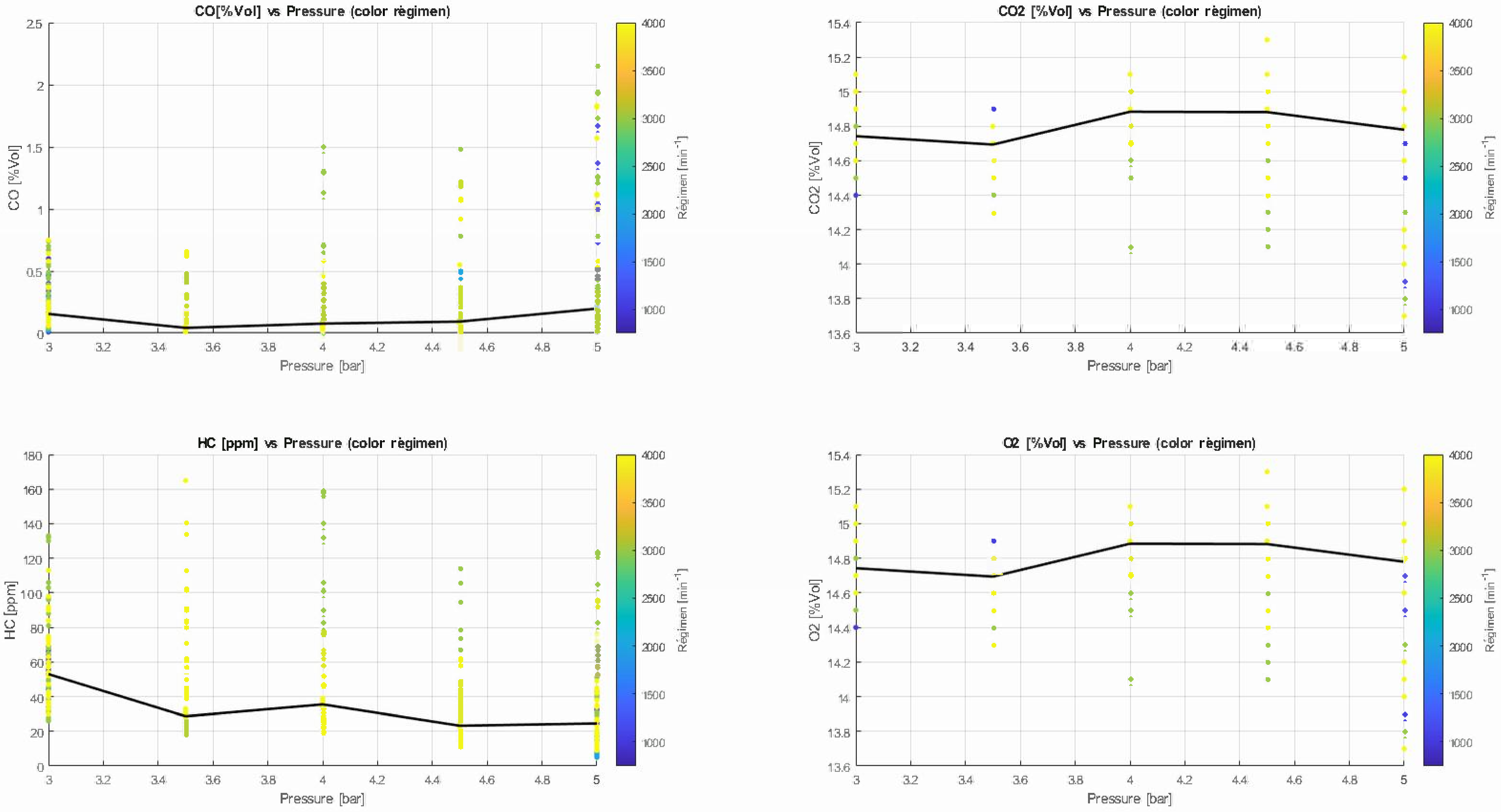

Figure S16. Emissions

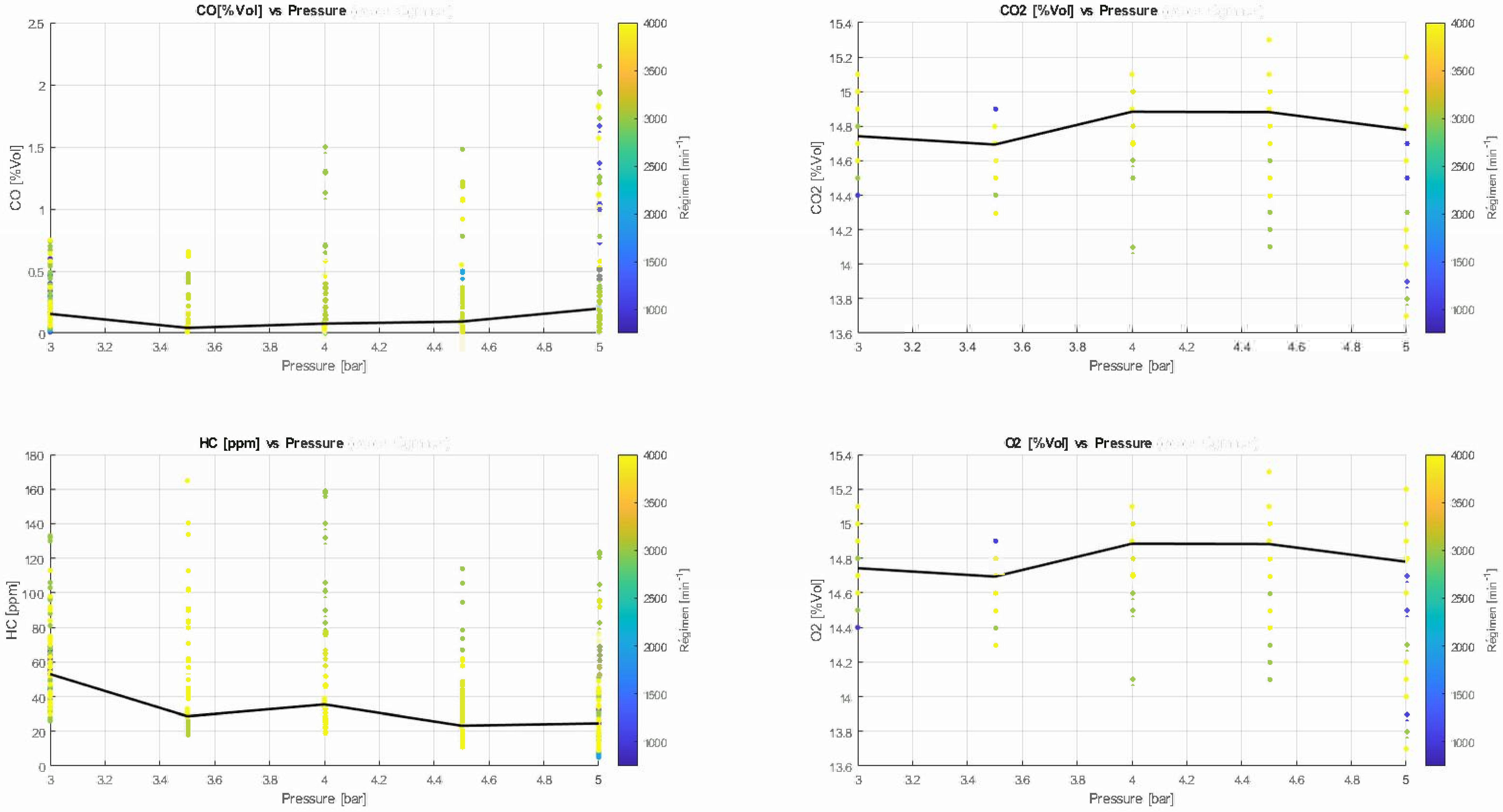

Figure S17. Engine load vs. main variables change

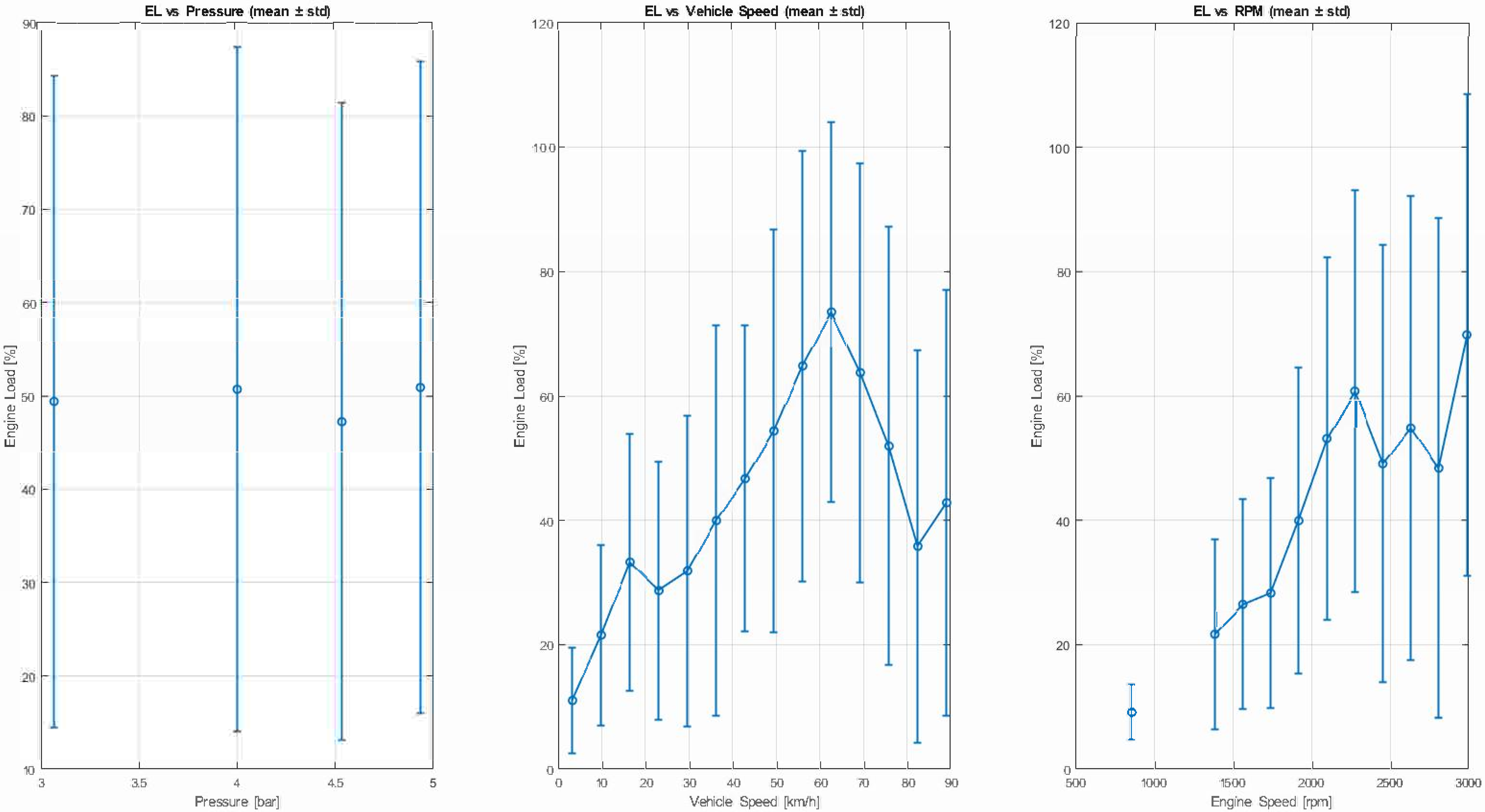

Figure S18. Engine load vs. pressure

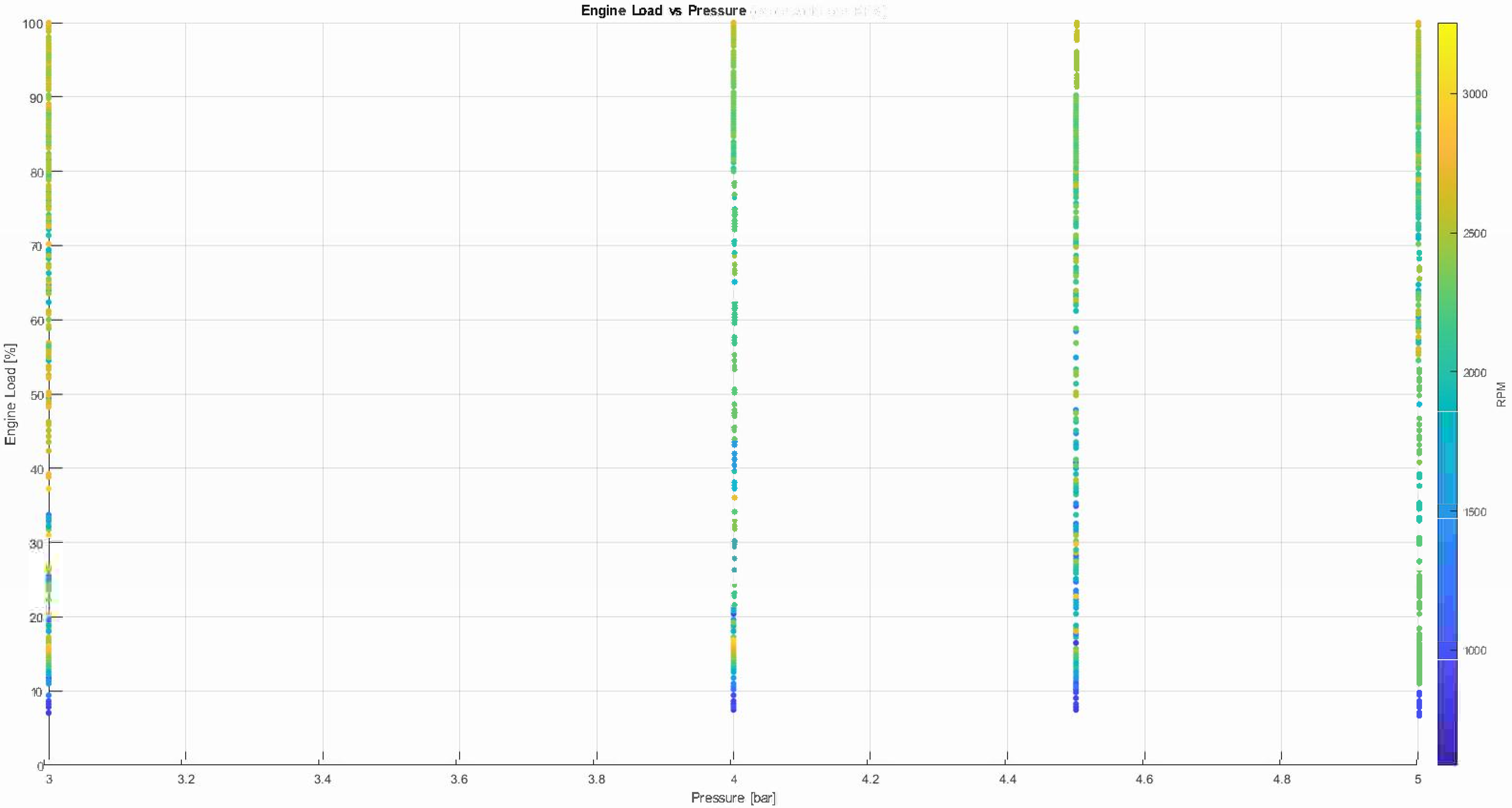

Figure S19. Forces vs. pressure

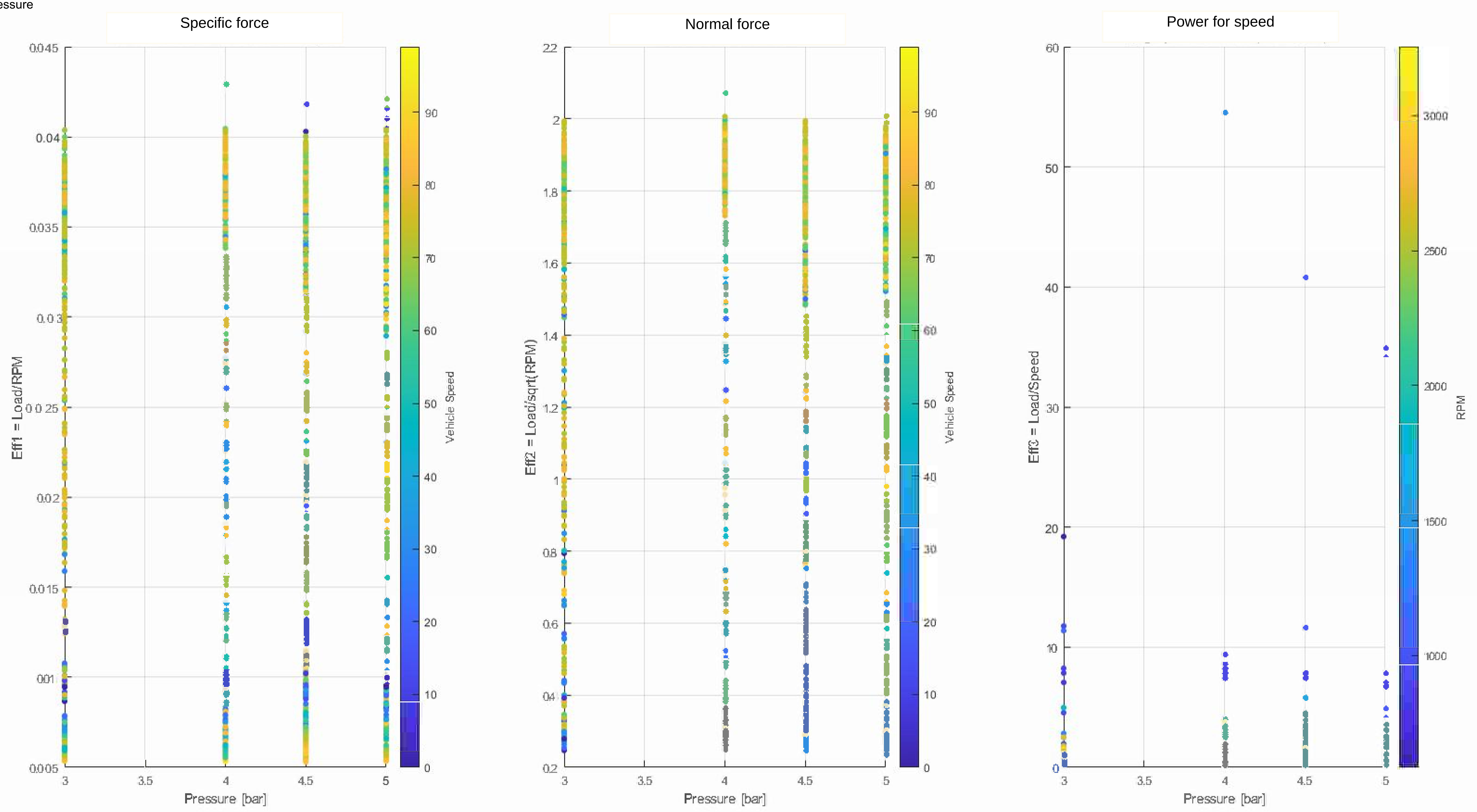

Figure S20. HC vs. pressure

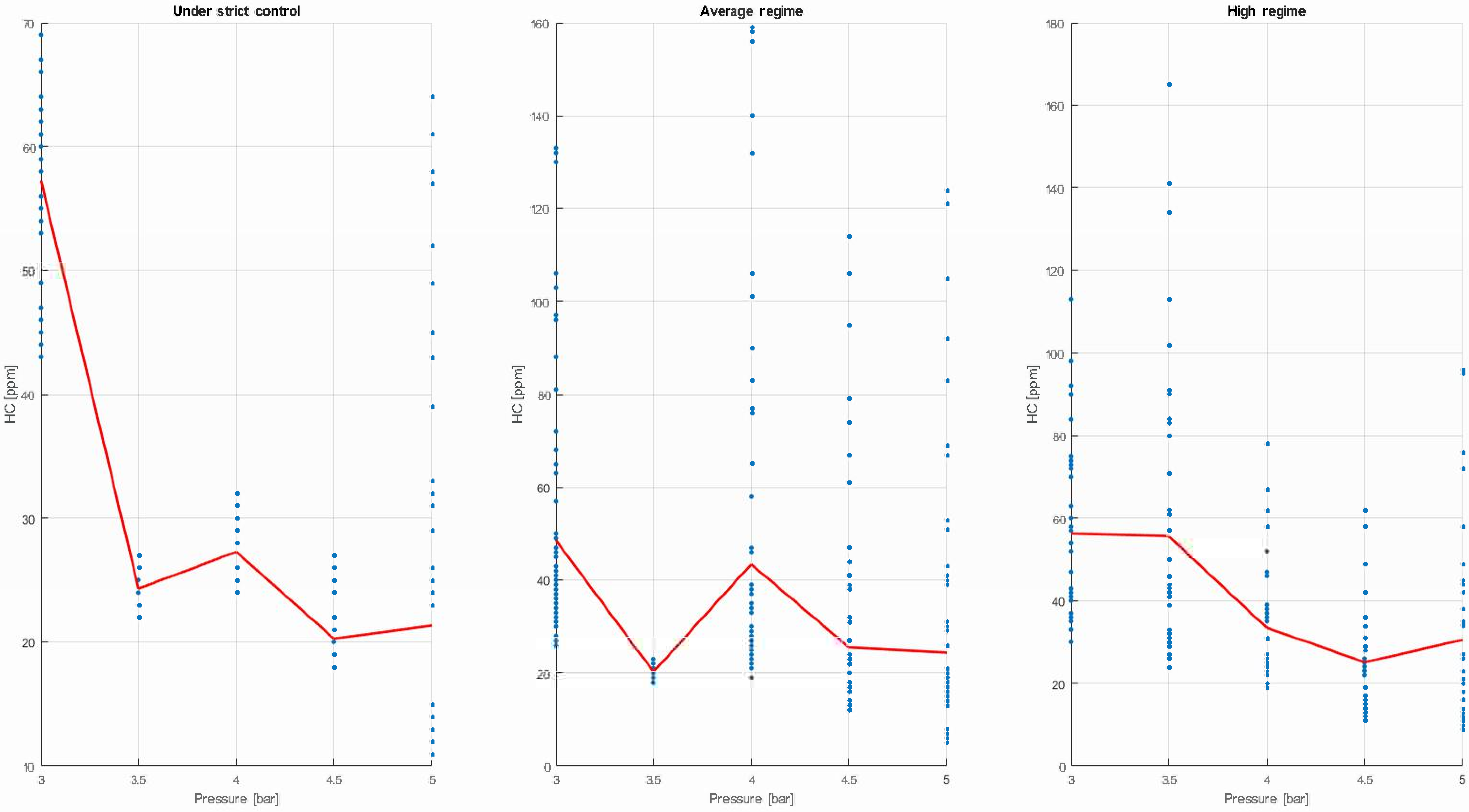

Figure S21. Optimal pressure

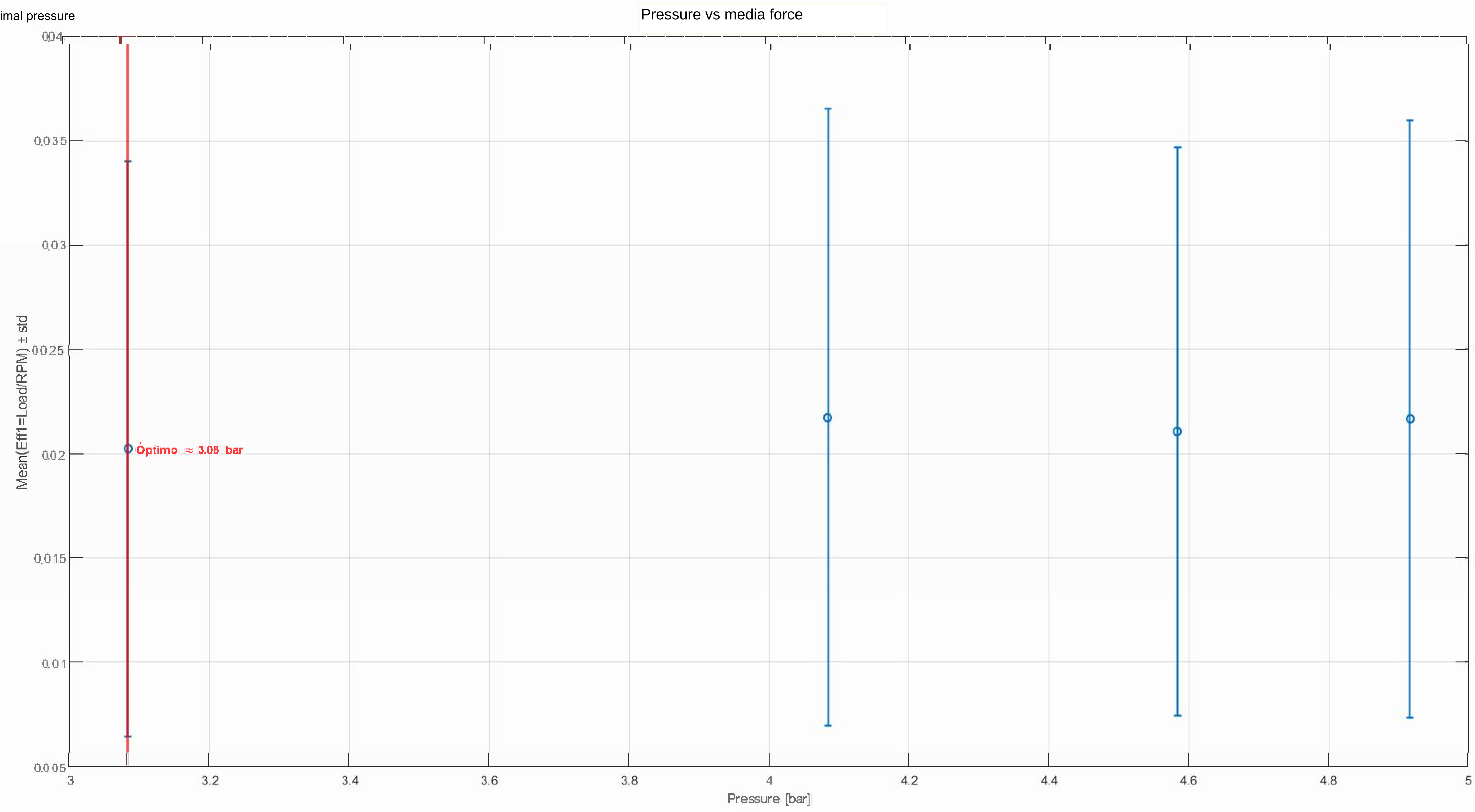

Figure S22. Pressure vs. rpm vs. engine load

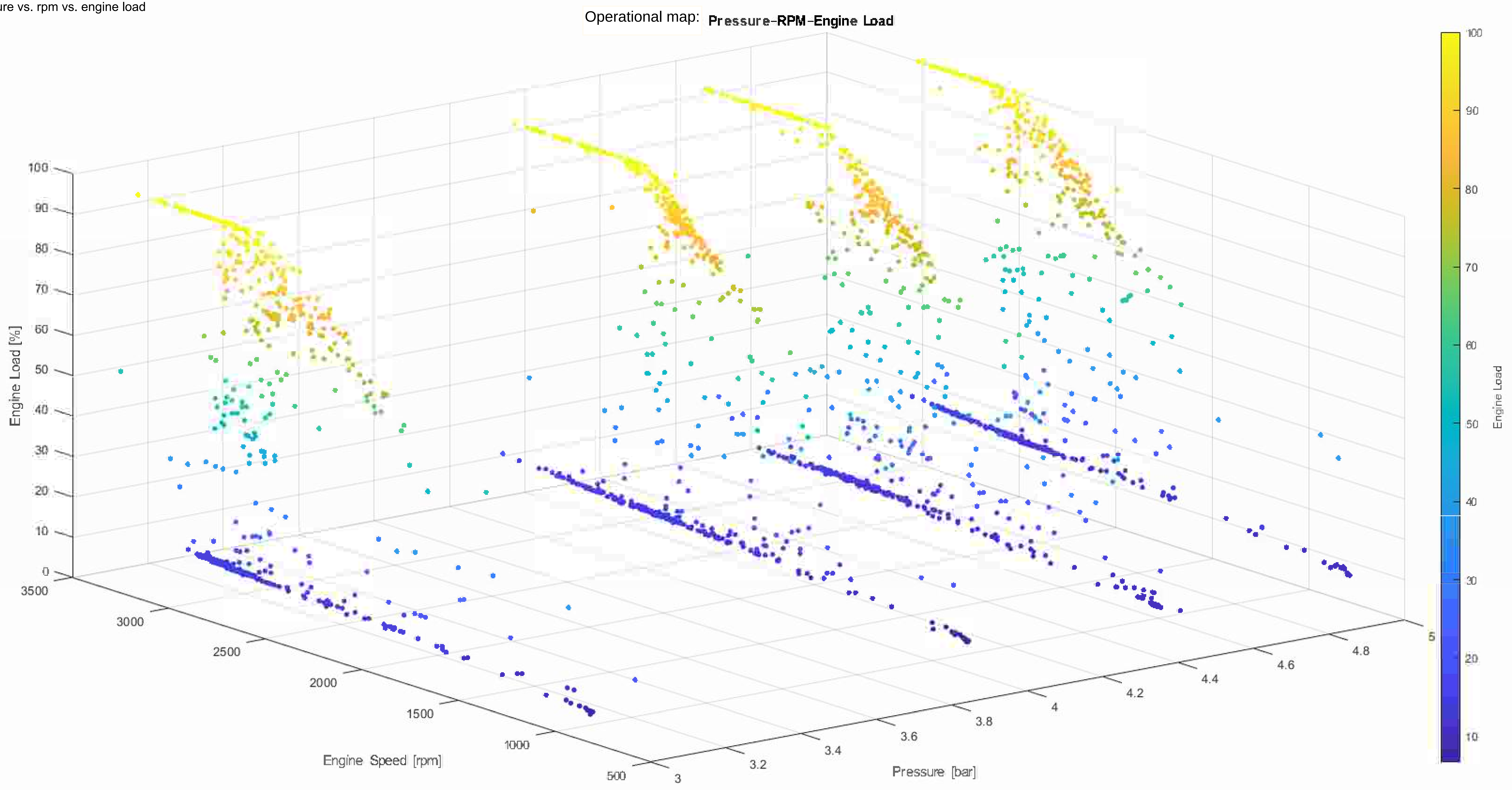

Supplement: Supplementary file 11 — Supplementary Material 11 [file 41598_2026_41765_MOESM11_ESM.pdf]
